# Supplementary material for: Effects of integrated economic and health interventions with women’s groups on health-related knowledge, behaviours and outcomes in low-income and middle-income countries: a systematic review protocol
Source: BMJ Open. 2021 Jul 12;11(7):e046856. doi: 10.1136/bmjopen-2020-046856 (PMC8276287; doi:10.1136/bmjopen-2020-046856)
Supplement: Supplementary data [file bmjopen-2020-046856supp001.pdf]

# Effects of integrated economic and health interventions with women's groups on health-related knowledge, behaviours and outcomes in low-and middle-income countries: a systematic review protocol

Sapna Desai, Kala M. Mehta, Roopal Jyoti Singh, Allie K. Westley, Osasuyi Dirisu, Connie Wong, Thomas de Hoop, Gary L. Darmstadt

## Supplementary File

## Appendix 1. Electronic database search strategy

Appendix 1. Search strategy using PubMed

(Microfinance*[tw] OR microcredit*[TW] OR "micro-finance" OR "micro-credit" OR microloan* OR "micro-loan" OR microlending OR microinsurance OR "micro-insurance" OR "village bank" OR "savings group" OR "village banks" OR "savings groups" OR "self help group" OR "self help groups" OR "self-help group" OR "self-help groups" OR "self-help groups"[Mesh] OR "community mobilization"[TW]OR "community mobilization"[TW] OR "social mobilisation" OR "social mobilization" OR "community mobilisation"[TW] OR "financial support"[Mesh]) AND ("female"[Mesh] OR women OR "women"[Mesh] OR "mothers"[Mesh] OR "women's group" OR "women's groups" OR "women's group-based") AND (((health[TW] OR "health"[Mesh] OR "infections"[Mesh] OR infection[tw] OR infectious[tw] OR "sanitation"[Mesh] OR "waste management"[Mesh] OR toilet*[tw] OR "toilet facilities"[Mesh] OR "psychiatry"[Mesh] OR "mental health"[Mesh]OR wellbeing OR "well-being" OR "HIV infections"[Mesh] OR "HIV"[TW] OR "AIDS"[tw] OR "breastfeeding" OR "breast feeding"[Mesh] OR "breast feeding" OR "acquired immunodeficiency syndrome" OR "malaria" OR violen*[tw] OR "maternal health" OR "neonatal health" OR "child health" OR "family health" OR "infant health"[Mesh] OR "sexual health" OR "women's health"[Mesh] OR "maternal health"[Mesh] OR "newborn health" OR "safety" OR "family planning" OR immunization OR "vaccination"[Mesh] OR nutrition OR feeding[tiab] OR "malnutrition"[Mesh] OR "medication adherence"[Mesh] OR "health behaviors" OR "health behavior"[Mesh] OR "health behaviours" OR "health behaviour"[Mesh] OR "child growth"[Mesh] OR "water-borne"[tw] OR "vector-borne"[tw] OR "health awareness" OR "sexually transmitted diseases"[Mesh] OR "STD"[tiab] OR "sexually transmitted diseases"[tw]OR "school health" OR "Delivery, Obstetric"[Mesh]) AND (intervention*[tiab] OR program*[tiab] OR promotion[tiab] OR train*[tiab] OR education[TW] OR campaign*[TW] OR service*[Tiab] OR control*[tiab])) OR "health promotion"[Mesh] OR "health education"[Mesh]OR "health knowledge, attitudes, practice"[Mesh]) AND ("developing country"[tw] OR "developing countries"[tw] OR "developing nation"[tw] OR "developing nations"[tw] OR "developing population"[tw] OR "developing populations"[tw] OR "developing world"[tw] OR "less developed country"[tw] OR "less developed countries"[tw]OR "less developed nation"[tw] OR "less developed nations"[tw] OR "less developed population"[tw] OR "less developed populations"[tw] OR "less developed world"[tw] OR "lesser developed country"[tw] OR "lesser developed countries"[tw] OR "lesser developed nation"[tw]OR "lesser developed nations"[tw] OR "lesser developed population"[tw] OR "lesser developed populations"[tw] OR "lesser developed world"[tw] OR "under developed country"[tw] OR "under developed countries"[tw] OR "under developed nation"[tw] OR "under developed nations"[tw] OR "under developed population"[tw] OR "under developed populations"[tw] OR "under developed world"[tw] OR "underdeveloped country"[tw] OR "underdeveloped countries"[tw] OR "underdeveloped nation"[tw] OR "underdeveloped nations"[tw] OR "underdeveloped population"[tw] OR "underdeveloped populations"[tw] OR "underdeveloped world"[tw] OR "middle income country"[tw] OR "middle income countries"[tw] OR "middle income nation"[tw] OR "middle income nations"[tw] OR "middle income population"[tw] OR "middle income populations"[tw] OR "low income country"[tw] OR "low income countries"[tw] OR "low income nation"[tw] OR "low income nations"[tw] OR "low income population"[tw] OR "low income populations"[tw] OR "lower income country"[tw] OR "lower income countries"[tw] OR "lower income nation"[tw] OR "lower income nations"[tw] OR "lower income population"[tw] OR "lower income populations"[tw] OR "underserved country"[tw] OR "underserved countries"[tw] OR "underserved nation"[tw] OR "underserved nations"[tw] OR "underserved population"[tw] OR "underserved populations"[tw] OR "underserved world"[tw] OR "underserved country"[tw] OR "underserved countries"[tw] OR "underserved nation"[tw] OR "underserved nations"[tw] OR "underserved population"[tw] OR "underserved populations"[tw]OR "underserved world"[tw] OR "deprived country"[tw] OR "deprived countries"[tw] OR "deprived nation"[tw] OR "deprived nations"[tw] OR "deprived population"[tw] OR "deprived populations"[tw] OR "deprived world"[tw] OR "poor country"[tw] OR "poor countries"[tw]OR "poor nation"[tw] OR "poor nations"[tw] OR "poor population"[tw] OR "poor populations"[tw] OR "poor world"[tw] OR "poorer country"[tw] OR "poorer countries"[tw] OR "poorer nation"[tw] OR "poorer nations"[tw] OR "poorer population"[tw] OR "poorer populations"[tw]OR "poorer world"[tw] OR "developing economy"[tw] OR "developing economies"[tw] OR "less developed economy"[tw] OR "less developed economies"[tw] OR "lesser developed economy"[tw] OR "lesser developed economies"[tw] OR "under developed economy"[tw] OR "underdeveloped economies"[tw] OR "underdeveloped economy"[tw] OR "underdeveloped economies"[tw] OR "middle income economy"[tw] OR "middle income economies"[tw] OR "low income economy"[tw] OR "low income economies"[tw] OR "lower income economy"[tw] OR "lower income economies"[tw] OR "low gdp"[tw] OR "low gnp"[tw] OR "low gross domestic"[tw] OR "low gross national"[tw] OR "lower gdp"[tw] OR "lower gnp"[tw] OR "lower gross domestic"[tw] OR "lower gross national"[tw] OR "lmic"[tw] OR lmics[tw] OR "third world"[tw] OR "lamic country"[tw] OR "lami countries"[tw] OR "transitional country"[tw] OR "transitional countries"[tw] OR Africa[tw] OR Asia[tw] OR Caribbean[tw] OR West Indies[tw] OR South America[tw] OR Latin America[tw] OR Central America[tw] OR Afghanistan[tw] OR Albania[tw]OR Algeria[tw] OR Angola[tw] OR Antigua[tw] OR Barbuda[tw] OR Argentina[tw] OR Armenia[tw] OR Armenian[tw] OR Aruba[tw] OR Azerbaijan[tw] OR Bahrain[tw] OR Bangladesh[tw] OR Barbados[tw] OR Benin[tw] OR Byelarus[tw] OR

Byelorussian[tw] OR Belarus[tw] OR Belorussian[tw]OR Belorussia[tw] OR Belize[tw] OR Bhutan[tw] OR Bolivia[tw] OR Bosnia[tw] OR Herzegovina[tw] OR Hercegovina[tw] OR Botswana[tw] OR Brasil[tw] OR Brazil[tw] OR Bulgaria[tw] OR Burkina Faso[tw] OR Burkina Fasso[tw] OR Upper Volta[tw] OR Burundi[tw] OR Urundi[tw]OR Cambodia[tw] OR Khmer Republic[tw] OR Kampuchea[tw] OR Cameroon[tw] OR Cameroons[tw] OR Cameron[tw] OR Cape Verde[tw] OR Central African Republic[tw] OR Chad[tw] OR Chile[tw] OR China[tw] OR Colombia[tw] OR Comoros[tw] OR Comoro Islands[tw]OR Comores[tw] OR Mayotte[tw] OR Congo[tw] OR Zaire[tw] OR Costa Rica[tw] OR Cote d'Ivoire[tw] OR Ivory Coast[tw] OR Croatia[tw] OR Cuba[tw] OR Cyprus[tw] OR Czechoslovakia[tw] OR Czech Republic[tw] OR Slovakia[tw] OR Slovak Republic[tw] OR Djibouti[tw] OR French Somaliland[tw] OR Dominica[tw] OR Dominican Republic[tw] OR East Timor[tw] OR East Timur[tw] OR Timor Leste[tw] OR Ecuador[tw] OR Egypt[tw] OR United Arab Republic[tw] OR El Salvador[tw] OR Eritrea[tw] OR Estonia[tw] OR Ethiopia[tw] OR Fiji[tw] OR Gabon[tw]OR Gabonese Republic[tw] OR Gambia[tw] OR Gaza[tw] OR Georgia Republic[tw] OR Georgian Republic[tw] OR Ghana[tw] OR Gold Coast[tw] OR Greece[tw] OR Grenada[tw] OR Guatemala[tw] OR Guinea[tw] OR Guam[tw] OR Guiana[tw] OR Guyana[tw] OR Haiti[tw] OR Honduras[tw]OR Hungary[tw] OR India[tw] OR Maldives[tw] OR Indonesia[tw] OR Iran[tw] OR Iraq[tw] OR Isle of Man[tw] OR Jamaica[tw] OR Jordan[tw] OR Kazakhstan[tw] OR Kazakh[tw] OR Kenya[tw] OR Kiribati[tw] OR Korea[tw] OR Kosovo[tw] OR Kyrgyzstan[tw] OR Kirghizia[tw]OR Kyrgyz Republic[tw] OR Kirghiz[tw] OR Kirgizstan[tw] OR "Lao PDR"[tw] OR Laos[tw] OR Latvia[tw] OR Lebanon[tw] OR Lesotho[tw] OR Basutoland[tw] OR Liberia[tw] OR Libya[tw] OR Lithuania[tw] OR Macedonia[tw] OR Madagascar[tw] OR Malagasy Republic[tw] OR Malaysia[tw]OR Malaya[tw] OR Malay[tw] OR Sabah[tw] OR Sarawak[tw] OR Malawi[tw] OR Nyasaland[tw] OR Mali[tw] OR Malta[tw] OR Marshall Islands[tw] OR Mauritania[tw] OR Mauritius[tw] OR Agalega Islands[tw] OR Mexico[tw] OR Micronesia[tw] OR Middle East[tw] OR Moldova[tw]OR Moldovia[tw] OR Moldovian[tw] OR Mongolia[tw] OR Montenegro[tw] OR Morocco[tw] OR Ifni[tw] OR Mozambique[tw] OR Myanmar[tw] OR Myanma[tw] OR Burma[tw] OR Namibia[tw] OR Nepal[tw] OR Netherlands Antilles[tw] OR New Caledonia[tw] OR Nicaragua[tw] OR Niger[tw]OR Nigeria[tw] OR Northern Mariana Islands[tw] OR Oman[tw] OR Muscat[tw] OR Pakistan[tw] OR Palau[tw] OR Palestine[tw] OR Panama[tw] OR Paraguay[tw] OR Peru[tw] OR Philippines[tw] OR Philipines[tw] OR Phillipines[tw] OR Phillippines[tw] OR Poland[tw] OR Portugal[tw]OR Puerto Rico[tw] OR Romania[tw] OR Rumania[tw] OR Roumania[tw] OR Russia[tw] OR Russian[tw] OR Rwanda[tw] OR Ruanda[tw] OR “Saint Kitts”[tw] OR “St Kitts and Nevis”[tw] OR Saint Lucia[tw] OR St Lucia[tw] OR Saint Vincent[tw] OR St Vincent[tw] OR Grenadines[tw]OR Samoa[tw] OR Samoan Islands[tw] OR Navigator Island[tw] OR Navigator Islands[tw] OR Sao Tome[tw] OR Saudi Arabia[tw] OR Senegal[tw] OR Serbia[tw] OR Montenegro[tw] OR Seychelles[tw] OR Sierra Leone[tw] OR Slovenia[tw] OR Sri Lanka[tw] OR Ceylon[tw] OR Solomon Islands[tw] OR Somalia[tw] OR Sudan[tw] OR Suriname[tw] OR Surinam[tw] OR Swaziland[tw] OR Syria[tw] OR Tajikistan[tw] OR Tadzhikistan[tw] OR Tadjikistan[tw] OR Tadzhik[tw] OR Tanzania[tw] OR Thailand[tw] OR Togo[tw] OR Togolese Republic[tw] OR Tonga[tw] OR Trinidad[tw] OR Tobago[tw] OR Tunisia[tw] OR Turkey[tw] OR Turkmenistan[tw] OR Turkmen[tw] OR Uganda[tw] OR Ukraine[tw] OR Uruguay[tw] OR USSR[tw] OR Soviet Union[tw] OR Union of Soviet Socialist Republics[tw] OR Uzbekistan[tw] OR Uzbek OR Vanuatu[tw] OR New Hebrides[tw] OR Venezuela[tw] OR Vietnam[tw] OR Viet Nam[tw] OR West Bank[tw] OR Yemen[tw] OR Yugoslavia[tw] OR Zambia[tw] OR Zimbabwe[tw] OR Rhodesia[tw] OR Developing Countries[Mesh:noexp] OR Africa[Mesh:noexp] OR Africa, Northern[Mesh:noexp] OR Africa South of the Sahara[Mesh:noexp] OR Africa, Central[Mesh:noexp] OR Africa, Eastern[Mesh:noexp] OR Africa, Southern[Mesh:noexp] OR Africa, Western[Mesh:noexp] OR Asia[Mesh:noexp] OR Asia, Central[Mesh:noexp] OR Asia, Southeastern[Mesh:noexp] OR Asia, Western[Mesh:noexp]OR Caribbean Region[Mesh:noexp] OR West Indies[Mesh:noexp] OR South America[Mesh:noexp] OR Latin America[Mesh:noexp] OR Central America[Mesh:noexp] OR Afghanistan[Mesh:noexp] OR Albania[Mesh:noexp] OR Algeria[Mesh:noexp] OR American Samoa[Mesh:noexp] OR Angola[Mesh:noexp]OR "Antigua and Barbuda"[Mesh:noexp] OR Argentina[Mesh:noexp] OR Armenia[Mesh:noexp] OR Azerbaijan[Mesh:noexp] OR Bahrain[Mesh:noexp] OR Bangladesh[Mesh:noexp] OR Barbados[Mesh:noexp] OR Benin[Mesh:noexp] OR Byelarus[Mesh:noexp] OR Belize[Mesh:noexp] OR Bhutan[Mesh:noexp]OR Bolivia[Mesh:noexp] OR Bosnia-Herzegovina[Mesh:noexp] OR Botswana[Mesh:noexp] OR Brazil[Mesh:noexp] OR Bulgaria[Mesh:noexp] OR Burkina Faso[Mesh:noexp] OR Burundi[Mesh:noexp] OR Cambodia[Mesh:noexp] OR Cameroon[Mesh:noexp] OR Cape Verde[Mesh:noexp] OR Central African Republic[Mesh:noexp] OR Chad[Mesh:noexp] OR Chile[Mesh:noexp] OR China[Mesh:noexp] OR Colombia[Mesh:noexp] OR Comoros[Mesh:noexp] OR Congo[Mesh:noexp] OR Costa Rica[Mesh:noexp] OR Cote d'Ivoire[Mesh:noexp] OR Croatia[Mesh:noexp] OR Cuba[Mesh:noexp]OR Cyprus[Mesh:noexp] OR Czechoslovakia[Mesh:noexp] OR Czech Republic[Mesh:noexp] OR Slovakia[Mesh:noexp] OR Djibouti[Mesh:noexp] OR "Democratic Republic of the Congo"[Mesh:noexp] OR Dominica[Mesh:noexp] OR Dominican Republic[Mesh:noexp] OR East Timor[Mesh:noexp]OR Ecuador[Mesh:noexp] OR Egypt[Mesh:noexp] OR El Salvador[Mesh:noexp] OR Eritrea[Mesh:noexp] OR Estonia[Mesh:noexp] OR Ethiopia[Mesh:noexp] OR Fiji[Mesh:noexp] OR Gabon[Mesh:noexp] OR Gambia[Mesh:noexp] OR "Georgia (Republic)"[Mesh:noexp] OR Ghana[Mesh:noexp]OR Greece[Mesh:noexp] OR Grenada[Mesh:noexp] OR Guatemala[Mesh:noexp] OR Guinea[Mesh:noexp] OR Guinea-Bissau[Mesh:noexp] OR Guam[Mesh:noexp] OR Guyana[Mesh:noexp] OR Haiti[Mesh:noexp] OR Honduras[Mesh:noexp] OR Hungary[Mesh:noexp] OR India[Mesh:noexp] OR Indonesia[Mesh:noexp]OR Iran[Mesh:noexp] OR Iraq[Mesh:noexp] OR Jamaica[Mesh:noexp] OR Jordan[Mesh:noexp] OR Kazakhstan[Mesh:noexp] OR Kenya[Mesh:noexp] OR Korea[Mesh:noexp] OR Kosovo[Mesh:noexp] OR Kyrgyzstan[Mesh:noexp] OR Laos[Mesh:noexp] OR Latvia[Mesh:noexp] OR Lebanon[Mesh:noexp]OR Lesotho[Mesh:noexp] OR Liberia[Mesh:noexp] OR Libya[Mesh:noexp] OR Lithuania[Mesh:noexp] OR Macedonia[Mesh:noexp] OR Madagascar[Mesh:noexp] OR Malaysia[Mesh:noexp] OR Malawi[Mesh:noexp] OR Mali[Mesh:noexp] OR Malta[Mesh:noexp] OR Mauritania[Mesh:noexp]OR Mauritius[Mesh:noexp] OR Mexico[Mesh:noexp] OR Micronesia[Mesh:noexp] OR Middle East[Mesh:noexp] OR Moldova[Mesh:noexp] OR Mongolia[Mesh:noexp] OR Montenegro[Mesh:noexp] OR Morocco[Mesh:noexp] OR Mozambique[Mesh:noexp] OR Myanmar[Mesh:noexp] OR Namibia[Mesh:noexp]OR Nepal[Mesh:noexp] OR Netherlands Antilles[Mesh:noexp] OR New Caledonia[Mesh:noexp] OR Nicaragua[Mesh:noexp] OR Niger[Mesh:noexp] OR Nigeria[Mesh:noexp] OR Oman[Mesh:noexp] OR Pakistan[Mesh:noexp] OR Palau[Mesh:noexp] OR Panama[Mesh:noexp] OR Papua New Guinea[Mesh:noexp]OR Paraguay[Mesh:noexp] OR Peru[Mesh:noexp] OR Philippines[Mesh:noexp] OR Poland[Mesh:noexp] OR Portugal[Mesh:noexp] OR Puerto Rico[Mesh:noexp] OR Romania[Mesh:noexp] OR Russia[Mesh:noexp] OR "Russia (Pre-1917)"[Mesh:noexp] OR Rwanda[Mesh:noexp] OR "Saint Kitts and Nevis"[Mesh:noexp] OR Saint Lucia[Mesh:noexp] OR "Saint Vincent and the Grenadines"[Mesh:noexp] OR Samoa[Mesh:noexp] OR Saudi Arabia[Mesh:noexp] OR Senegal[Mesh:noexp] OR Serbia[Mesh:noexp] OR Montenegro[Mesh:noexp] OR Seychelles[Mesh:noexp] OR Sierra Leone[Mesh:noexp] OR Slovenia[Mesh:noexp] OR Sri Lanka[Mesh:noexp] OR Somalia[Mesh:noexp] OR South Africa[Mesh:noexp] OR Sudan[Mesh:noexp] OR Suriname[Mesh:noexp] OR Swaziland[Mesh:noexp] OR Syria[Mesh:noexp] OR Tajikistan[Mesh:noexp] OR Tanzania[Mesh:noexp]OR

Thailand[Mesh:noexp] OR Togo[Mesh:noexp] OR Tonga[Mesh:noexp] OR "Trinidad and Tobago"[Mesh:noexp] OR Tunisia[Mesh:noexp] OR Turkey[Mesh:noexp] OR Turkmenistan[Mesh:noexp] OR Uganda[Mesh:noexp] OR Ukraine[Mesh:noexp] OR Uruguay[Mesh:noexp] OR USSR[Mesh:noexp]OR Uzbekistan[Mesh:noexp] OR Vanuatu[Mesh:noexp] OR Venezuela[Mesh:noexp] OR Vietnam[Mesh:noexp] OR Yemen[Mesh:noexp] OR Yugoslavia[Mesh:noexp] OR Zambia[Mesh:noexp] OR Zimbabwe[Mesh:noexp])



Desai S, *et al. BMJ Open* 2021; 11:e046856. doi: 10.1136/bmjopen-2020-046856

**Appendix 2.** Extraction sheet

| Appendix 2, Extraction terms | |
|---|---|
| **Background details** | First author surname<br>Year<br>Title<br>Country<br>Region<br>Intervention- Funding source<br>Evaluation- Funding source<br>Author email address |
| **Study Context** | Study location:<br>rural=1;<br>urban=2;<br>peri-urban=3;<br>mixed (urban/rural/peri-urban)=4<br>Background and history of groups<br>(who formed, trained and maintained the groups in the study areas)<br>Health system strength/indicators<br>(in author's words- the health systems setting, the important health indicators talked about in the study area)<br>Specific policy environment towards groups (NRLM, linkages with other devlopment programmes)<br>Others |
| **Hypothesis tested \|&\| Theory of Change** | What is the underlying hypothesis being tested in the paper?<br>What is the implicit or explicit theory of change?<br>Were any statistical analyses done to test the TOC?<br>Yes/ No- 1/0<br>What are the effect modifiers/ anticipated effect modifiers, if any<br>What SBC theory is used implicitly or explicitly? |
| **Intervention** | Describe the intervention and its components in the authors' own words<br>Intervention description- On field implementation details (Text from paper/ rewrite in brief)<br>What elements of the intervention were participatory (in authors' own words)<br>What is the average group size?<br>Who was the facilitator?<br>(1- Internal member, trained from within the group; 2- External facilitator, from NGO or Govt.)<br>Facilitator characteristics (education, residence, social group, sex, etc).<br>What training was given to the facilitator in authors' own words?<br>Were the facilitators given any monetary incentive? Y=1, N=0<br>Number of groups per facilitator (con) |



Desai S, *et al. BMJ Open* 2021; 11:e046856. doi: 10.1136/bmjopen-2020-046856

| | |
|---|---|
| | Number of groups per facilitator (exp)<br>intervention period?<br>Number of sessions<br>Is this a mixed method evaluation? Yes=1, No=2<br>Level of participation of members in intervention design and implementation (Arnstein): Informed/Consulted/Partners<br>Level of capability strengthening for health (Labonte and Laverack): Individual/Group/community/other<br>Intervention communication approach: Didactic/Interactive/Participatory<br>Category of group intervention (Gram et al): Class/Club/Collective |
| **Group details** | How often did groups meet?<br>Underlying group type:-<br>1= Microfinance groups<br>2= Open groups<br>3= Community based women only groups<br>4= Special population groups<br>Average age (and range) of members in groups<br>What is the duration of each meeting?<br>Population coverage of groups? (% coverage)<br>Group dissolution during the intervention period<br>What are the service linkages (health specific), if any? |
| **Intensity/ Coverage of Intervention** | Details on functioning of Groups in Control arm<br>Details on functioning of Groups in Intervention arm<br>Topics discussed in a regular (control) group meeting + length of discussion<br>Additional topics discussed in Intervention arm group meetings (Due to layering) + length of discussion<br>Frequency of add-on intervention<br>% members who attended the add-on intervention sessions<br>Exposure to the intervention (text from paper, if any)<br>Target group of intervention - does this align with group membership (age, demographic characteristics). In authors words if available |
| **Participant Inclusion Criterion** | Evaluation-<br>Participant eligibility, inclusion criteria [text]<br>Evaluation-<br>Participant eligibility, Exclusion criteria [text]<br>Intervention-<br>Participant eligibility, inclusion criteria [text]<br>Intervention-<br>Participant eligibility, Exclusion criteria [text] |
| **Evaluation details** | How would you characterise the evaluation on the efficacy/effectiveness spectrum? |

| | |
|---|---|
| | 1 = towards efficacy (implementation on small/medium scale by non-gov partners);<br>2 = implementation on small/medium scale by gov partners in conditions close to routine;<br>3 = implementation on large scale, through routine training systems by gov partners<br>Evaluation design (1= RCT,<br>2=Longitudinal Cohort,<br>3= Repeated Cross-sectional survey<br>4= Pre/ post evaluation design<br>5=Other (specify, eg. Type of Quasi experimental)<br>Nature of comparison (1=exp vs con; 2=before vs after intervention)<br>Is there a control group? Yes=1, No=2<br>Type of control (group with no intervention OR no group, with or without matching)<br>Is this a cross-sectional baseline/endline survey design? Yes=1, No=2<br>Please describe survey design [text] |
| **Cost effectiveness component** | Does the study include a cost effectiveness component?<br>Yes--1<br>No--2<br>Is the cost given a total cost figure, or separate cost given for each descriptive component of the intervention?<br>Total cost--1<br>Individual cost components--2<br>Cost effectiveness method used (text from paper)<br>Cost value (figure given in the paper with unit) |
| **Outcome areas** | MNCH<br>SRH and HIV<br>Family planning<br>General health/morbidity<br>Violence<br>Mental health<br>Nutrition<br>(Includes Breastfeeding)<br>Non-communicable diseases<br>Communicable diseases<br>Expenditure<br>Health insurance<br>Sanitation<br>Outcomes at group level or population level?<br>1- Group level<br>2- Population/ Community level<br>3. Sample members and non-members (from the community)<br>Non health outcomes, if any?<br>What is the primary outcome?<br>What are the secondary outcomes?<br>Does study differentiate between primary and secondary outcomes? Yes=1/no=0 |



Desai S, *et al. BMJ Open* 2021; 11:e046856. doi: 10.1136/bmjopen-2020-046856

| | |
|---|---|
| **Timing of data collection** | baseline<br>endline<br>Time between baseline and endline<br>other information (if any, write it in text) |
| **Data collection and statistical methods** | Sampling methods in the authors' own words<br>Is the analysis by Intention-to-treat> (yes=1, no=0)<br>Confounders assessed/adjusted for?<br>Unit of clustering<br><br>Clustering at --<br>1: geographical level, 2: clusters formed using specific characteristics (other than geography)<br>Number and size of clusters<br>Adjusted for clustering in analysis? Yes=1/no=0<br>Does the study have a sample size calculation? Yes=1, No=0<br>Does the study have a published protocol or registration with details of outcomes and analysis methods? Yes=1, No=0<br>Randomisation method, if any<br>Blinding process, in authors' own words (i.e. were any or all of persons who did random allocation, data collectors, and analysts blind to exposure/cont rolallocation?).<br>N eligible participants (exp vs control or before/after)<br>Loss to follow-up/Attrition (individual)<br>Method of assessment (write name of questionnaire/describe method)<br>e.g specific scales used like PHQ, CESD etc or anthropometric or biometric measurements taken<br>Statistical method (the name of analysis) |
| **Results** | mean age- separate baseline and endline if appropriate<br>Literacy - separate baseline and endline if appropriate<br>% of participants belonging to marginalised groups (con) - separate baseline and endline if appropriate |
| | N eligible participants (exp vs control or before/after) |
| **Results: Primary Outcomes** | Results - Mean (SD) in exp/con or before/after participants<br>Results - Rate in exp/con or before/after participants<br>Results - Prevalence in exp/con or before/after participants<br>Results - incidence in exp/con or before/after participants<br>Results -Odds Ratio (95% CI) for exp/con or before/after participants specify adjusted and unadjusted<br>Results - Rate ratio (95% CI) for exp/con or before/after participants specify adjusted and unadjusted<br>Results; Beta coefficient (95% CI) for exp/con or before/after participants specify adjusted and unadjusted<br>Results: difference-in-difference (DID) (difference in % points)- control v/s intervention before vs after |
| | Results; P-value for difference between groups |
| **Results: Secondary Outcomes** | Results: report all results on secondary outcomes with effect sizes (95% CI) and p-value - use ITT results, not sub-group analyses - |

| | |
|---|---|
| | Highlight significant findings (CI does not include 1) in red or with asterisk<br>Notes on secondary outcomes |
| **Qualitative Results and Appraisal** | Qualitative sample<br>Qualitative data collection methods<br>Qualitative results<br>Clarity of the research question<br>Explanation of the context<br>Sampling<br>Reflexivity of the account<br>Clear account of the methods used for data collection and analysis<br>Amount of data pertinent to the review question |
| **Authors' Own Account Of Study Limitations** | Notes on bias |
| **Risk of Bias assessment** | ROB-2 and ROBINS |
| **Barriers And Enablers To Intervention Achieving Impact** | Enablers for the intervention in the authors words<br>Barriers for the intervention in the authors words<br>Reviewers interpretation of barriers and enablers. |
| **Layering specific Barriers and Enablers** | Barriers to layering the intervention<br>Enablers to layering the intervention |
| **Process Evaluation** | Does this study include a process evaluation<br>Is the process evaluation quantitative, qualitative or mixed methods (give citation here) |
| **Additional information, if any** | Notes/ Remarks |



Desai S, *et al. BMJ Open* 2021; 11:e046856. doi: 10.1136/bmjopen-2020-046856
