# Supplementary material for: Effects of integrated economic and health interventions with women’s groups on health-related knowledge, behaviours and outcomes in low-income and middle-income countries: a systematic review protocol
Source: BMJ Open. 2021 Jul 12;11(7):e046856. doi: 10.1136/bmjopen-2020-046856 (PMC8276287; doi:10.1136/bmjopen-2020-046856)
Supplement: Author's manuscript [file bmjopen-2020-046856.draft_revisions.pdf]

BMJ Open

BMJ Open is committed to open peer review. As part of this commitment we make the peer review history of every article we publish publicly available.

When an article is published we post the peer reviewers' comments and the authors' responses online. We also post the versions of the paper that were used during peer review. These are the versions that the peer review comments apply to.

The versions of the paper that follow are the versions that were submitted during the peer review process. They are not the versions of record or the final published versions. They should not be cited or distributed as the published version of this manuscript.

BMJ Open is an open access journal and the full, final, typeset and author-corrected version of record of the manuscript is available on our site with no access controls, subscription charges or pay-per-view fees (http://bmjopen.bmj.com).

If you have any questions on BMJ Open's open peer review process please email info.bmjopen@bmj.com

BMJ Open

Effects of integrated economic and health interventions with women's groups on health-related knowledge, behaviours and outcomes in low-and middle-income countries: a systematic review protocol

Journal:	*BMJ Open*
Manuscript ID	bmjopen-2020-046856
Article Type:	Protocol
Date Submitted by the Author:	11-Nov-2020
Complete List of Authors:	Desai, Sapna; Population Council India<br>Mehta, Kala; University of California San Francisco<br>Singh, Roopal; Population Council India<br>Westley, Allie; Stanford University<br>Dirisu, Osasuyi; Population Council Nigeria<br>Wong, Connie; Lane Medical Library; Stanford University School of Medicine<br>De Hoop, Thomas; American Institutes for Research<br>Darmstadt, Gary; Stanford University School of Medicine, Pediatrics
Keywords:	PUBLIC HEALTH, HEALTH ECONOMICS, International health services < HEALTH SERVICES ADMINISTRATION & MANAGEMENT, Health economics < HEALTH SERVICES ADMINISTRATION & MANAGEMENT

SCHOLARONE™
Manuscripts

BMJ

I, the Submitting Author has the right to grant and does grant on behalf of all authors of the Work (as defined in the below author licence), an exclusive licence and/or a non-exclusive licence for contributions from authors who are: i) UK Crown employees; ii) where BMJ has agreed a CC-BY licence shall apply, and/or iii) in accordance with the terms applicable for US Federal Government officers or employees acting as part of their official duties; on a worldwide, perpetual, irrevocable, royalty-free basis to BMJ Publishing Group Ltd ("BMJ") its licensees and where the relevant Journal is co-owned by BMJ to the co-owners of the Journal, to publish the Work in this journal and any other BMJ products and to exploit all rights, as set out in our licence.

The Submitting Author accepts and understands that any supply made under these terms is made by BMJ to the Submitting Author unless you are acting as an employee on behalf of your employer or a postgraduate student of an affiliated institution which is paying any applicable article publishing charge ("APC") for Open Access articles. Where the Submitting Author wishes to make the Work available on an Open Access basis (and intends to pay the relevant APC), the terms of reuse of such Open Access shall be governed by a Creative Commons licence – details of these licences and which Creative Commons licence will apply to this Work are set out in our licence referred to above.

Other than as permitted in any relevant BMJ Author's Self Archiving Policies, I confirm this Work has not been accepted for publication elsewhere, is not being considered for publication elsewhere and does not duplicate material already published. I confirm all authors consent to publication of this Work and authorise the granting of this licence.

For peer review only

Effects of integrated economic and health interventions with women's groups on health-related knowledge, behaviours and outcomes in low-and middle-income countries: a systematic review protocol

Short title: Effects of women's groups with integrated economic and health interventions

For peer review only

Sapna Desai,[1] Kala M. Mehta,[2] Roopal Jyoti Singh,[1] Allie K. Westley, [3] Osasuyi Dirisu, [4] Connie Wong, [5] Thomas de Hoop,[6] Gary L. Darmstadt[7]

[1]Population Council, New Delhi, India, sdesai@popcouncil.org, ORCID #

[2]University of California, San Francisco, CA, USA, kmehta23@stanford.edu

[3] Stanford University, Stanford, CA, USA, awestley@stanford.edu

[4] Population Council, Abuja, Nigeria, odirisu@popcouncil.org

[5]Lane Medical Library, Stanford University School of Medicine, Stanford, CA, USA, hongnei@stanford.edu

[6]American Institutes for Research, Washington DC, USA, tdehoop@air.org

[7]Department of Pediatrics, Stanford University School of Medicine, Stanford, CA, USA, gdarmsta@stanford.edu

Corresponding author: Gary L. Darmstadt, MD, MS, Department of Pediatrics, Stanford University School of Medicine, 1701 Page Mill Road, Palo Alto, CA 94304, USA, gdarmsta@stanford.edu

Word Count: Abstract, 253, body, 2362.

For peer review only - http://bmjopen.bmj.com/site/about/guidelines.xhtml

Abstract

Introduction

Economic groups such as microfinance or self-help groups (SHGs) are widely implemented in low and middle-income countries (LMICs). Women's groups are voluntary groups which aim to improve members' well-being through activities such as joint savings, credit, livelihoods development and/or health activities. Health interventions are increasingly added on to existing women's economic groups as a public health intervention for women and their families. Here we present the protocol for a mixed-methods systematic review we will conduct of the evidence on integrated economic and health interventions upon women's groups to assess whether and how they improve health-related knowledge, behaviour and outcomes in LMICs.

Methods and analysis

We will search six electronic databases for published and grey literature, along with manual searches and consultation. The review will include (i) randomised trials and non-randomised quasi-experimental studies of intervention effects of integrated economic and health interventions delivered through women's groups in LMICs, and (ii) sibling studies that examine factors related to intervention content, context, implementation processes and costs. We will appraise risk of bias and study quality using standard tools. High and moderate-quality studies will be grouped by health domain and synthesised without meta-analysis. Qualitative evidence will be thematically synthesised and integrated into the quantitative synthesis using a matrix approach.

Ethics and dissemination

This protocol was reviewed and deemed exempt by the institutional review board at the American Institutes for Research. Findings will be shared through peer-reviewed publication and disseminated with programme implementers and policymakers engaged with women's groups.

For peer review only - http://bmjopen.bmj.com/site/about/guidelines.xhtml

Registration

This protocol is registered as PROSPERO CRD42020199998.

Strengths and limitations of this study

- We outline the protocol for a systematic review of current global literature exploring a highly important method of health education delivery for women.
- This convergent mixed-methods review will utilise robust methods to integrate quantitative evidence on intervention effectiveness with qualitative studies to provide programme and policy-relevant evidence on the effects of integrated women's groups interventions on health outcomes.
- The review will include sibling studies such as process evaluations identified through a comprehensive search of published and grey literature and will summarise intervention content, context, implementation processes, facilitating factors and barriers, and costs.
- The review will include a comprehensive risk-of-bias assessment to critically appraise study quality.
- The review is limited by lack of meta-analysis, given heterogeneity of reported outcomes.

For peer review only

Introduction

Women's groups come together in many settings in low and middle-income countries (LMICs) to improve women's opportunities for livelihoods and financial security.[1] Women's self-help groups (SHGs) and collectives have developed and spread throughout India for several decades; the National Rural Livelihoods Mission (NRLM) has reached over 70 million households with microfinance and livelihoods interventions through SHGs since 2011.[2] Similarly, Village Savings and Loan Associations (VSLA) and other savings groups have operated for in many parts of Africa for over three decades.[3] Economic women's groups typically focus on savings, access to credit and livelihoods to improve financial inclusion and economic outcomes. A recent systematic review found that livelihoods groups have small but positive effects on consumption and moderate effects on savings, but did not find effects on income, asset ownership or labour force participation.[1] The review suggests that livelihoods groups may have more impact when combined with investments in human capital, including health. Previous reports of the effect of combined microfinance and health interventions, though not all group-based, suggest some evidence of improvements in health behaviours amongst participants.[4, 5]

Governments and other stakeholders increasingly support integrating (or "layering") additional activities onto livelihoods-based groups, most commonly with health interventions. Combined economic and health interventions implemented through groups have been evaluated for impacts on a range of health outcomes including violence against women,[6, 7] health-promoting behaviours[8] and malnutrition amongst women and children.[9] Despite growing policy interest, there has been no systematic review of the evidence on combined economic and health interventions through women's groups to inform ongoing and future programmes. This paper presents the protocol, following Preferred Reporting Items for Systematic Review and Meta-analysis Protocols (PRISMA-P) guidelines,[10] for a review we will conduct which aims to address this gap through synthesising quantitative and qualitative evidence on the health effects of

For peer review only - http://bmjopen.bmj.com/site/about/guidelines.xhtml

combined group-based economic and health interventions and implementation factors that contributed to achieving intended outcomes. We aim to answer two primary questions: 1) What is the effect of combined economic and health, nutrition and/or sanitation interventions delivered through women's groups on health outcomes amongst women and children in LMICs, compared to single-purpose interventions with women's groups or to no group intervention, and 2) What factors related to intervention content, context and implementation processes are enablers or barriers to achieving health outcomes?

Methods

Study design

Study inclusion criteria

This review is a mixed-methods convergent review in which synthesis of quantitative effects and qualitative evidence for factors in intervention implementation will be combined within the review.[11] Our review will include all trials – both randomised controlled trials and non-randomised, quasi-experimental studies – on integrated economic and health interventions through groups and which include a control or comparison arm. Non-randomised studies of interventions (NRSI) include stronger and weaker designs.[12] Criteria for inclusion of NRSI will be ability to address selection bias either through the inclusion of a baseline measurement of the outcome of interest or other relevant confounding factors, or through intervention allocation rules that enable the use of regression discontinuity designs or natural experiments.[13, 14] Additional inclusion criteria include that the intervention was implemented in a LMIC according to World Bank classification[15] and the study was published between January 1, 2000 and May 31, 2020 in the peer-reviewed or grey literature. We will also include studies which are linked to the included trials, including process evaluations, qualitative studies and cost or cost-effectiveness studies, i.e. "trial sibling studies."[16]

Study exclusion criteria

For peer review only - http://bmjopen.bmj.com/site/about/guidelines.xhtml

Studies with the following characteristics will be excluded: 1) No qualitative or quantitative data were collected to evaluate the effect of an intervention, for example the study only provides observational descriptions of groups; 2) The treatment group only implements an economic or health intervention, i.e. the intervention is not an integrated approach; 3) No comparison group was included; 4) No baseline measurement was reported in a non-randomised, quasi-experimental study; 5) An independent studies on women's groups was not linked to an impact evaluation (non-sibling studies); and 6) The study was published prior to January 1, 2000.

Participants

Participant inclusion criteria

For this review, we define an eligible woman's group as a group that was constituted for women to regularly exchange information, support or goods (e.g. savings) and plan individual and/or collective action. Mixed-sex groups will be included if there were 51% or more women. We will include closed groups and groups constituted for women that were also open to others to attend meetings. We define adult women to be 18 years of age or more.

Participant exclusion criteria

Our review will exclude men's only groups, family groups, select committees of community representatives, professional groups and political associations. We will also exclude adolescent girls' groups, as a recent systematic review examines these groups and the role of integrated interventions compared to singular approaches,[17] as well as groups that did not have a joint purpose or objective to meet regularly, such as short-term training sessions.

Types of interventions

Intervention inclusion criteria

For peer review only

The review will focus on integrated economic and health/sanitation/nutrition interventions delivered through women's' groups. Groups may be pre-existing in the study areas or may have been set up as part of the group intervention. Interventions will include combinations of health and/or nutrition and/or sanitation components with an economic intervention aimed to improve financial inclusion, income, consumption or livelihoods, such as microfinance, entrepreneurial skills training or collective enterprise development.

Intervention exclusion criteria

We will not examine economic women's groups that did not have a health, sanitation or nutrition intervention component (defined below), or women's groups that solely had health interventions, without economic interventions, such as mothers' groups. If the intervention was not described in sufficient detail to categorise the type of intervention, it will not be included.

Comparisons

Comparisons will include groups with non-intervention controls and 'usual' practice. We will include evaluations that compared the integrated intervention to either no group or to a singular model (economic or health). For example, groups with an economic component but no health, sanitation, or nutrition intervention can serve as the comparison group.

Outcomes

Condition or domain being studied will include any health, nutrition or sanitation-related knowledge, behaviours and outcomes across all domains consistent with the World Health Organisation definition of health as 'a state of complete physical, mental and social wellbeing.'[18] Domains will include (but are not limited to): maternal, newborn and child health; sexual and reproductive health; nutrition; infectious and vector-borne disease; non-communicable disease; mental health; violence against women; water, sanitation and hygiene; health services utilisation;

For peer review only

and health expenditure. The domain of maternal health, for example, may include both objectively measured health outcomes such as maternal mortality and anaemia, along with behaviours during the perinatal period and knowledge of postnatal care. Infectious and vector-borne disease may include outcomes related to vector density or incidence of malaria, along with behaviours such as sleeping under a bednet or seeking treatment for fever and knowledge of symptoms of malaria or dengue.

For peer review only

Information sources

Electronic databases

We will search the following six electronic research databases: Pubmed, Scopus, Embase, Web of Science, PsycINFO and Social Sciences Citation Index (SSCI).

Other sources

Studies will also be obtained for the same time period from reference lists of included studies, reference lists of systematic reviews on the effects of women's groups, the 3ie Database of Impact Evaluations and Evidence Consortium on Women's Groups repository, and Google Scholar. In addition, we will contact study authors to identify process evaluations or qualitative studies conducted in conjunction with an impact evaluation and consult with experts familiar with interventions in Francophone Africa and Latin/South America to recommend inclusion of French or Spanish language articles.

Search strategy

The search strategy for PubMed is in Appendix 1. This strategy will be adapted to the other electronic databases by the library scientist on our team (CW), with any modifications reported in the review manuscript.

For peer review only - http://bmjopen.bmj.com/site/about/guidelines.xhtml

Data management

The titles and abstracts retrieved by electronic searches will be exported to a systematic review application called COVIDENCE. Reference management for full text will be conducted using Endnote.

Selection strategy

Two reviewers will independently screen abstracts and titles. The reviewers will not be blind to the author or journal information. The abstracts of papers that are in a language other than English will be translated using Google Translate. If considered eligible or eligibility is unclear, professional translation of the full paper will be undertaken. The full texts of articles will be obtained for all potentially eligible studies for further examination. For all excluded articles, the primary reason for exclusion will be recorded and documented in the excluded studies table. Discrepancies between the two review authors regarding study eligibility will be resolved by discussion and consensus and if necessary, by a third reviewer.

Data collection process

Two investigators will independently collect data from articles selected for full text review using an extraction form developed by the authors and piloted before use. Discrepancies between reviewers regarding data extraction will be resolved by discussion and consensus, and if necessary, by contacting authors.

w only

Data items

The following information will be extracted into COVIDENCE and an Excel file (see the full list of data extraction items in Appendix 2): Publication details: authors, year and journal; study context and study design; hypothesis tested and theory of change; participant inclusion criteria and demographic characteristics; characteristics of the intervention, including the duration, intervention

For peer review only - http://bmjopen.bmj.com/site/about/guidelines.xhtml

strategies, intensity and coverage; outcomes (primary and secondary); data collection and statistical methods, including sample size; findings of process evaluations and cost effectiveness; source(s) of research funding and potential conflicts of interest; and study limitations in the authors' own words. Attempts will be made to contact the corresponding authors of included studies if there are specific queries regarding the items for extractions that are unavailable in the published manuscript.

Outcomes and prioritisation

We will extract data on all health outcomes, as detailed above. Primary outcomes, as identified by the authors, will be prioritised in reporting. If authors have not distinguished between primary and secondary outcomes, we will report all outcomes. In addition, we will extract data on non-health outcomes reported by authors, such as on women's empowerment or economic outcomes.

Risk of bias in individual studies

We will appraise risk of bias in trials using the Cochrane guides for randomised controlled trials (Cochrane ROB-2) and for assessing risk of bias in non-randomised studies of interventions (ROBINS-1).[19, 20] We will examine risk of bias for primary outcomes. If a primary outcome is not specified, we will utilise the outcome for which sample size was determined. Process evaluations will be appraised using an adapted version of an eight-item tool developed by the EPPI Centre, with additions specific to group-based interventions.[21, 22]

Data synthesis

We will first summarise characteristics of all trials with their risk of bias appraisal, grouped by health domain. Given the anticipated heterogeneity of health outcomes, we do not plan to conduct a meta-analysis. We will synthesise findings from trials with low or moderate risk of bias in tabular form and in the narrative text. We will present forest plots by health domain without a summary

For peer review only - http://bmjopen.bmj.com/site/about/guidelines.xhtml
For peer review only

measure if possible, and potentially present harvest plots to compare effects by factors related to the health intervention approach. Synthesis methods and results will be presented according to the Synthesis without meta-analysis (SWiM) guidelines.[23]

Findings from process evaluations and other qualitative studies will be analysed separately in a thematic synthesis.[24] We will integrate the synthesis on intervention effects with the qualitative synthesis by juxtaposing findings in a matrix.[25] If consistent programme theories emerge across interventions, we may conduct a synthesis by programme theory.

Meta-biases

We will plot study sample size against effect size to evaluate whether these are skewed and asymmetrical in the presence of publication bias and other biases.[26] In addition, the risk of bias assessment tools will identify whether studies indicate selective reporting of outcomes, which we will summarise across studies if possible.

Confidence in cumulative evidence

The overall quality of evidence on outcomes will be presented using the Grades of Recommendation, Assessment, Development and Evaluation (GRADE) criteria.[27] This method will include an evaluation of within-study risk of bias (methodological quality), directness of evidence, heterogeneity, precision of effect estimates and risk of publication bias.

Patient and public involvement

There will be no patient or public involvement in the conduct or dissemination of the results of this study.

Ethics and dissemination

For peer review only - http://bmjopen.bmj.com/site/about/guidelines.xhtml

For peer review only

Our findings will be submitted for peer-reviewed publication. Deviations from the study protocol will be noted in the manuscript. Findings will be disseminated through conference presentations and shared with both programme implementers and policymakers engaged in interventions with women's groups, as well as on the website of the Evidence Consortium on Women's Groups (www.womensgroupevidence.org). No primary data collection will be undertaken and the institutional review board at the American Institutes for Research in Washington, D.C, determined that the review was exempt from human subjects review.

Discussion

This proposed review will contribute to a growing body of literature regarding how different types of women's groups may contribute to improved health outcomes.[4, 28. 29] The focus on integrated interventions is novel and also timely, as several countries and organisations are considering scaling these interventions in a variety of geographic contexts. Building a systematically aggregated, critically appraised knowledge base will aid in discerning the up-to-date evidence base regarding the health effects of integrated economic and health interventions. This review will also report on different types of women's groups, building on existing typologies,[30] to advance understanding on how interventions may be integrated. Systematically synthesizing the evidence for health effects as well as facilitating factors in creating health benefits will aid understanding of which integrated women's groups interventions will potentially work, for whom, in what context, and with which pre-conditions. Further, the incorporation of a mixed-methods approach and qualitative synthesis of implementation evidence will enable better understanding of how to implement women's group interventions effectively in various contexts.[25]

It is the intention that this review will be published in a peer-reviewed journal, will be presented at relevant global health and development conferences and the learnings will be disseminated to key-stakeholders working with or funding work with women's groups in LMIC contexts. Not only will this

For peer review only - http://bmjopen.bmj.com/site/about/guidelines.xhtml

work be important in and of itself, it will also shape and define several new research questions that will be critical to the field as it moves forward.

Authors' contributions

SD, GLD and TdH conceptualised the review. SD and KM drafted the protocol, with critical inputs from all authors. CW developed the search methodology. RJS, OD and AW developed and piloted extraction sheets. All authors reviewed and approved the final protocol.

Funding statement

This work was supported by the Bill & Melinda Gates Foundation in support of the Evidence Consortium on Women's Groups, through grant number OPP1201417. The funder provided input to the protocol, but the senior author had responsibility for the content and the decision to publish the protocol.

Competing interest statement

The authors have no competing interests. If studies conducted by any author are included in the review, this will be noted in the manuscript for publication

For peer review only - http://bmjopen.bmj.com/site/about/guidelines.xhtml

For peer review only

References

1. Barooah B, Chinoy S, Dubey P, et al. Improving and sustaining livelihoods through group-based interventions: Mapping the evidence (3ie Evidence Gap Map Report 13). *New Delhi, India: International Initiative for Impact Evaluation (3ie)* 2019
2. NRLM. Deendayal Antyodaya Yojana - National Rural Livelihoods Mission 2011 [Available from: https://aajeevika.gov.in/en accessed 15.9.2020.
3. Hendricks L, Chidiac S. Village savings and loans: A pathway to financial inclusion for Africa's poorest households. *Enterprise Development and Microfinance* 2011;22(2):134-46.
4. Orton L, Pennington A, Nayak S, et al. Group-based microfinance for collective empowerment: a systematic review of health impacts. *Bulletin of the world health organization* 2016;94(9):694.
5. Lorenzetti LM, Leatherman S, Flax VL. Evaluating the effect of integrated microfinance and health interventions: an updated review of the evidence. *Health policy and planning* 2017;32(5):732-56.
6. Pronyk PM, Hargreaves JR, Kim JC, et al. Effect of a structural intervention for the prevention of intimate-partner violence and HIV in rural South Africa: a cluster randomised trial. *The lancet* 2006;368(9551):1973-83.
7. Jejeebhoy SJ, Santhya K, Acharya R, et al. Empowering women and addressing violence against them through self-help groups (SHGs): Population Council New Delhi, India 2017.
8. Flax VL, Negerie M, Ibrahim AU, et al. Integrating group counseling, cell phone messaging, and participant-generated songs and dramas into a microcredit program increases Nigerian women's adherence to international breastfeeding recommendations. *The Journal of nutrition* 2014;144(7):1120-24.
9. Raghunathan K, Kumar N, Gupta S, et al. Learning together: Experimental evidence on the impact of group-based nutrition interventions in rural Bihar: Intl Food Policy Res Inst 2020.
10. Shamseer L, Moher D, Clarke M, Ghersi D, Liberati A, Petticrew M, Shekelle P, Stewart L, PRISMA-P Group. Preferred reporting items for systematic review and meta-analysis protocols (PRISMA-P) 2015: elaboration and explanation. BMJ. 2015 Jan 2;349(jan02 1):g7647.
11. Noyes J, Booth A, Cargo M, et al. Qualitative evidence. *Cochrane Handbook for Systematic Reviews of Interventions* 2019:525-45.
12. Reeves BC, Wells GA, Waddington H. Quasi-experimental study designs series—paper 5: a checklist for classifying studies evaluating the effects on health interventions—a taxonomy without labels. *Journal of clinical epidemiology* 2017;89:30-42.
13. Cook TD, Wong VC. Better quasi-experimental practice. *The handbook of social research* 2008:134-65.
14. Shadish WR. Randomized controlled studies and alternative designs in outcome studies: Challenges and opportunities. *Research on Social Work Practice* 2011;21(6):636-43.
15. WorldBank. World Bank Country and Lending Groups 2020 [Available from: https://datahelpdesk.worldbank.org/knowledgebase/articles/906519-world-bank-country-and-lending-groups.
16. Moore GF, Audrey S, Barker M, et al. Process evaluation of complex interventions: Medical Research Council guidance. *bmj* 2015;350
17. Haberland NA, McCarthy KJ, Brady M. A systematic review of adolescent girl program implementation in low-and middle-income countries: Evidence gaps and insights. *Journal of Adolescent Health* 2018;63(1):18-31.
18. Organization WH. Preamble to the Constitution of WHO as adopted by the International Health Conference, 1946, 1946:100.

For peer review only

19. Higgins JP, Thomas J, Chandler J, et al. Cochrane handbook for systematic reviews of interventions: John Wiley & Sons 2019.
20. Sterne JA, Hernan MA, Reeves BC, et al. ROBINS-I: a tool for assessing risk of bias in non-randomised studies of interventions. *BMJ* 2016;355:i4919. doi: 10.1136/bmj.i4919 [published Online First: 2016/10/14]
21. Cargo M, Harris J, Pantoja T, et al. Cochrane Qualitative and Implementation Methods Group guidance series—paper 4: methods for assessing evidence on intervention implementation. *Journal of clinical epidemiology* 2018;97:59-69.
22. Rees R, Oliver K, Woodman J, et al. Children's views about obesity, body size, shape and weight: a systematic review. *EPPI-Centre, Institute of Education, University of London* 2009
23. Campbell M, McKenzie JE, Sowden A, et al. Synthesis without meta-analysis (SWiM) in systematic reviews: reporting guideline. *bmj* 2020;368
24. Thomas J, Harden A. Methods for the thematic synthesis of qualitative research in systematic reviews. *BMC medical research methodology* 2008;8(1):45.
25. Harden A, Thomas J, Cargo M, et al. Cochrane Qualitative and Implementation Methods Group guidance series—paper 5: methods for integrating qualitative and implementation evidence within intervention effectiveness reviews. *Journal of clinical epidemiology* 2018;97:70-78.
26. Sterne JA, Egger M. Funnel plots for detecting bias in meta-analysis: guidelines on choice of axis. *Journal of clinical epidemiology* 2001;54(10):1046-55.
27. Schünemann HJ, Higgins JP, Vist GE, et al. Completing 'Summary of findings' tables and grading the certainty of the evidence. *Cochrane Handbook for Systematic Reviews of Interventions* 2019:375-402.
28. Kumar N, Scott S, Menon P, et al. Pathways from women's group-based programs to nutrition change in South Asia: A conceptual framework and literature review. *Global food security* 2018;17:172-85.
29. Desai S, Misra M, Das A, et al. Community interventions with women's groups to improve women's and children's health in India: a mixed-methods systematic review of effects, enablers and barriers. BMJ Global Health Forthcoming 2020.
30. Gram L, Desai S, Prost A. Classroom, club or collective? Three types of community- based group intervention and why they matter for health. *BMJ Glob Health (Forthcoming)* 2020

For peer review only

Appendix 1. Electronic database search strategy

Appendix 1. Search strategy using PubMed

(Microfinance*[tw] OR microcredit*[TW] OR “micro-finance” OR “micro-credit” OR microloan* OR “micro-loan” OR microlending OR microinsurance OR “micro-insurance” OR “village bank” OR “savings group” OR “village banks” OR “savings groups” OR “self help group” OR “self help groups” OR “self-help group” OR “self-help groups” OR “self-help groups”[Mesh] OR “community mobilization”[TW]OR “community mobilization”[TW] OR “social mobilisation” OR “social mobilization” OR “community mobilisation”[TW] OR “financial support”[Mesh]) AND (“female”[Mesh] OR women OR “women”[Mesh] OR “mothers”[Mesh] OR “women’s group” OR “women’s groups” OR “women’s group-based”) AND (((health[TW] OR “health”[Mesh] OR “infections”[Mesh] OR infection[tw] OR infectious[tw] OR “sanitation”[Mesh] OR “waste management”[Mesh] OR toilet*[tw] OR “toilet facilities”[Mesh] OR “psychiatry”[Mesh] OR “mental health”[Mesh]OR wellbeing OR “well-being” OR “HIV infections”[Mesh] OR “HIV”[TW] OR “AIDS”[tw] OR “breastfeeding” OR “breast feeding”[Mesh] OR “breast feeding” OR “acquired immunodeficiency syndrome” OR “malaria” OR violen*[tw] OR “maternal health” OR “neonatal health” OR “child health” OR “family health” OR “infant health”[Mesh] OR “sexual health” OR “women’s health”[Mesh] OR “maternal health”[Mesh] OR “newborn health” OR “safety” OR “family planning” OR immunization OR “vaccination”[Mesh] OR nutrition OR feeding[tiab] OR “malnutrition”[Mesh] OR “medication adherence”[Mesh] OR “health behaviors” OR “health behavior”[Mesh] OR “health behaviours” OR “health behaviour”[Mesh] OR “child growth”[Mesh] OR “water-borne”[tw] OR “vector-borne”[tw] OR “health awareness” OR “sexually transmitted diseases”[Mesh] OR “STD”[tiab] OR “sexually transmitted diseases”[tw]OR “school health” OR “Delivery, Obstetric”[Mesh]) AND (intervention*[tiab] OR program*[tiab] OR promotion[tiab] OR train*[tiab] OR education[TW] OR campaign*[TW] OR service*[Tiab] OR control*[tiab])) OR “health promotion”[Mesh] OR “health education”[Mesh]OR “health knowledge, attitudes, practice”[Mesh]) AND ("developing country"[tw] OR "developing countries"[tw] OR "developing nation"[tw] OR "developing nations"[tw] OR "developing population"[tw] OR "developing populations"[tw] OR "developing world"[tw] OR "less developed country"[tw] OR "less developed countries"[tw]OR "less developed nation"[tw] OR "less developed nations"[tw] OR "less developed population"[tw] OR "less developed populations"[tw] OR "less developed world"[tw] OR "lesser developed country"[tw] OR "lesser developed countries"[tw] OR "lesser developed nation"[tw]OR "lesser developed nations"[tw] OR "lesser developed population"[tw] OR "lesser developed populations"[tw] OR "lesser developed world"[tw] OR "under developed country"[tw] OR "under developed countries"[tw] OR "under developed nation"[tw] OR "under developed nations"[tw] OR "under developed population"[tw] OR "under developed populations"[tw] OR "under developed world"[tw] OR "underdeveloped country"[tw] OR "underdeveloped countries"[tw] OR "underdeveloped nation"[tw] OR "underdeveloped nations"[tw] OR "underdeveloped population"[tw] OR "underdeveloped populations"[tw] OR "underdeveloped world"[tw] OR "middle income country"[tw] OR "middle income countries"[tw] OR "middle income nation"[tw] OR "middle income nations"[tw] OR "middle income population"[tw] OR "middle income populations"[tw] OR "low income country"[tw] OR "low income countries"[tw] OR "low income nation"[tw] OR "low income nations"[tw] OR "low income population"[tw] OR "low income populations"[tw] OR "lower income country"[tw] OR "lower income countries"[tw] OR "lower income nation"[tw] OR "lower income nations"[tw] OR "lower income population"[tw] OR "lower income populations"[tw] OR "underserved country"[tw] OR "underserved countries"[tw] OR "underserved nation"[tw] OR "underserved nations"[tw] OR "underserved population"[tw] OR "underserved populations"[tw] OR "underserved world"[tw] OR "underserved country"[tw] OR "underserved countries"[tw] OR "underserved nation"[tw] OR "underserved nations"[tw] OR "underserved population"[tw] OR "underserved populations"[tw]OR "underserved world"[tw] OR "deprived country"[tw] OR "deprived countries"[tw] OR "deprived nation"[tw] OR "deprived nations"[tw] OR "deprived population"[tw] OR "deprived populations"[tw] OR "deprived world"[tw] OR "poor country"[tw] OR "poor countries"[tw]OR "poor nation"[tw] OR "poor nations"[tw] OR "poor population"[tw] OR "poor populations"[tw] OR "poor world"[tw] OR "poorer country"[tw] OR "poorer countries"[tw] OR "poorer nation"[tw] OR "poorer nations"[tw] OR "poorer population"[tw] OR "poorer populations"[tw]OR "poorer world"[tw] OR "developing economy"[tw] OR "developing economies"[tw] OR "less developed economy"[tw] OR "less developed economies"[tw] OR "lesser developed economy"[tw] OR "lesser developed economies"[tw] OR "under developed economy"[tw] OR "underdeveloped economies"[tw] OR "underdeveloped economy"[tw] OR "underdeveloped economies"[tw] OR "middle income economy"[tw] OR "middle income economies"[tw] OR "low income economy"[tw] OR "low income economies"[tw] OR "lower income economy"[tw] OR "lower income economies"[tw] OR "low gdp"[tw] OR "low gnp"[tw] OR "low gross domestic"[tw] OR "low gross national"[tw] OR "lower gdp"[tw] OR "lower gnp"[tw] OR "lower gross domestic"[tw] OR "lower gross national"[tw] OR "lmic"[tw] OR lmics[tw] OR "third world"[tw] OR "lamic country"[tw] OR "lami countries"[tw] OR "transitional country"[tw] OR "transitional countries"[tw] OR Africa[tw] OR Asia[tw] OR Caribbean[tw] OR West Indies[tw] OR South America[tw] OR Latin America[tw] OR Central America[tw] OR Afghanistan[tw] OR Albania[tw]OR Algeria[tw] OR Angola[tw] OR Antigua[tw] OR Barbuda[tw] OR Argentina[tw] OR Armenia[tw] OR Armenian[tw] OR Aruba[tw] OR Azerbaijan[tw] OR Bahrain[tw] OR Bangladesh[tw] OR Barbados[tw] OR Benin[tw] OR Byelarus[tw] OR Byelorussian[tw] OR Belarus[tw] OR Belorussian[tw]OR Belorussia[tw] OR Belize[tw] OR Bhutan[tw] OR Bolivia[tw] OR Bosnia[tw] OR Herzegovina[tw] OR Hercegovina[tw] OR Botswana[tw] OR Brasil[tw] OR Brazil[tw] OR Bulgaria[tw] OR Burkina Faso[tw] OR Burkina Fasso[tw] OR Upper Volta[tw] OR Burundi[tw] OR Urundi[tw]OR Cambodia[tw] OR Khmer Republic[tw] OR Kampuchea[tw] OR Cameroon[tw] OR Cameroons[tw] OR Cameron[tw] OR Cape Verde[tw] OR Central African Republic[tw] OR Chad[tw] OR Chile[tw] OR China[tw] OR Colombia[tw] OR Comoros[tw] OR Comoro Islands[tw]OR Comores[tw] OR Mayotte[tw] OR Congo[tw] OR Zaire[tw] OR Costa Rica[tw] OR Cote d'Ivoire[tw] OR Ivory Coast[tw] OR Croatia[tw] OR Cuba[tw] OR Cyprus[tw] OR Czechoslovakia[tw] OR Czech Republic[tw] OR Slovakia[tw] OR Slovak Republic[tw] OR Djibouti[tw] OR French Somaliland[tw] OR Dominica[tw] OR Dominican Republic[tw] OR East Timor[tw] OR East Timur[tw] OR Timor Leste[tw] OR Ecuador[tw] OR Egypt[tw] OR United Arab Republic[tw] OR El Salvador[tw] OR Eritrea[tw] OR Estonia[tw] OR Ethiopia[tw] OR Fiji[tw] OR Gabon[tw]OR Gabonese Republic[tw] OR Gambia[tw] OR Gaza[tw] OR Georgia Republic[tw] OR Georgian Republic[tw] OR Ghana[tw] OR Gold Coast[tw] OR Greece[tw] OR Grenada[tw] OR Guatemala[tw] OR Guinea[tw] OR Guam[tw] OR Guiana[tw] OR Guyana[tw] OR Haiti[tw] OR Honduras[tw]OR Hungary[tw] OR India[tw] OR Maldives[tw] OR Indonesia[tw] OR Iran[tw] OR Iraq[tw] OR Isle of Man[tw] OR Jamaica[tw] OR Jordan[tw] OR Kazakhstan[tw] OR Kazakh[tw] OR Kenya[tw] OR Kiribati[tw] OR Korea[tw] OR Kosovo[tw] OR Kyrgyzstan[tw] OR Kirghizia[tw]OR Kyrgyz Republic[tw] OR Kirghiz[tw] OR Kirgizstan[tw] OR "Lao PDR"[tw] OR Laos[tw] OR Latvia[tw] OR Lebanon[tw] OR Lesotho[tw] OR Basutoland[tw] OR Liberia[tw] OR Libya[tw] OR Lithuania[tw] OR Macedonia[tw] OR Madagascar[tw] OR Malagasy Republic[tw] OR Malaysia[tw]OR Malaya[tw] OR Malay[tw] OR Sabah[tw] OR Sarawak[tw] OR Malawi[tw] OR Nyasaland[tw] OR Mali[tw] OR Malta[tw] OR Marshall Islands[tw] OR Mauritania[tw] OR Mauritius[tw] OR Agalega Islands[tw] OR Mexico[tw] OR Micronesia[tw] OR Middle East[tw] OR Moldova[tw]OR Moldovia[tw] OR Moldovian[tw] OR Mongolia[tw] OR Montenegro[tw] OR Morocco[tw] OR Ifni[tw] OR Mozambique[tw]

For peer review only - http://bmjopen.bmj.com/site/about/guidelines.xhtml

OR Myanmar[tw] OR Myanma[tw] OR Burma[tw] OR Namibia[tw] OR Nepal[tw] OR Netherlands Antilles[tw] OR New Caledonia[tw] OR Nicaragua[tw] OR Niger[tw]OR Nigeria[tw] OR Northern Mariana Islands[tw] OR Oman[tw] OR Muscat[tw] OR Pakistan[tw] OR Palau[tw] OR Palestine[tw] OR Panama[tw] OR Paraguay[tw] OR Peru[tw] OR Philippines[tw] OR Philipines[tw] OR Phillipines[tw] OR Phillippines[tw] OR Poland[tw] OR Portugal[tw]OR Puerto Rico[tw] OR Romania[tw] OR Rumania[tw] OR Roumania[tw] OR Russia[tw] OR Russian[tw] OR Rwanda[tw] OR Ruanda[tw] OR "Saint Kitts"[tw] OR "St Kitts and Nevis"[tw] OR Saint Lucia[tw] OR St Lucia[tw] OR Saint Vincent[tw] OR St Vincent[tw] OR Grenadines[tw]OR Samoa[tw] OR Samoan Islands[tw] OR Navigator Island[tw] OR Navigator Islands[tw] OR Sao Tome[tw] OR Saudi Arabia[tw] OR Senegal[tw] OR Serbia[tw] OR Montenegro[tw] OR Seychelles[tw] OR Sierra Leone[tw] OR Slovenia[tw] OR Sri Lanka[tw] OR Ceylon[tw] OR Solomon Islands[tw] OR Somalia[tw] OR Sudan[tw] OR Suriname[tw] OR Surinam[tw] OR Swaziland[tw] OR Syria[tw] OR Tajikistan[tw] OR Tadzhikistan[tw] OR Tadjikistan[tw] OR Tadzhik[tw] OR Tanzania[tw] OR Thailand[tw] OR Togo[tw] OR Togolese Republic[tw] OR Tonga[tw] OR Trinidad[tw] OR Tobago[tw] OR Tunisia[tw] OR Turkey[tw] OR Turkmenistan[tw] OR Turkmen[tw] OR Uganda[tw] OR Ukraine[tw] OR Uruguay[tw] OR USSR[tw] OR Soviet Union[tw] OR Union of Soviet Socialist Republics[tw] OR Uzbekistan[tw] OR Uzbek OR Vanuatu[tw] OR New Hebrides[tw] OR Venezuela[tw] OR Vietnam[tw] OR Viet Nam[tw] OR West Bank[tw] OR Yemen[tw] OR Yugoslavia[tw] OR Zambia[tw] OR Zimbabwe[tw] OR Rhodesia[tw] OR Developing Countries[Mesh:noexp] OR Africa[Mesh:noexp] OR Africa, Northern[Mesh:noexp] OR Africa South of the Sahara[Mesh:noexp] OR Africa, Central[Mesh:noexp] OR Africa, Eastern[Mesh:noexp] OR Africa, Southern[Mesh:noexp] OR Africa, Western[Mesh:noexp] OR Asia[Mesh:noexp] OR Asia, Central[Mesh:noexp] OR Asia, Southeastern[Mesh:noexp] OR Asia, Western[Mesh:noexp]OR Caribbean Region[Mesh:noexp] OR West Indies[Mesh:noexp] OR South America[Mesh:noexp] OR Latin America[Mesh:noexp] OR Central America[Mesh:noexp] OR Afghanistan[Mesh:noexp] OR Albania[Mesh:noexp] OR Algeria[Mesh:noexp] OR American Samoa[Mesh:noexp] OR Angola[Mesh:noexp]OR "Antigua and Barbuda"[Mesh:noexp] OR Argentina[Mesh:noexp] OR Armenia[Mesh:noexp] OR Azerbaijan[Mesh:noexp] OR Bahrain[Mesh:noexp] OR Bangladesh[Mesh:noexp] OR Barbados[Mesh:noexp] OR Benin[Mesh:noexp] OR Byelarus[Mesh:noexp] OR Belize[Mesh:noexp] OR Bhutan[Mesh:noexp]OR Bolivia[Mesh:noexp] OR Bosnia-Herzegovina[Mesh:noexp] OR Botswana[Mesh:noexp] OR Brazil[Mesh:noexp] OR Bulgaria[Mesh:noexp] OR Burkina Faso[Mesh:noexp] OR Burundi[Mesh:noexp] OR Cambodia[Mesh:noexp] OR Cameroon[Mesh:noexp] OR Cape Verde[Mesh:noexp] OR Central African Republic[Mesh:noexp] OR Chad[Mesh:noexp] OR Chile[Mesh:noexp] OR China[Mesh:noexp] OR Colombia[Mesh:noexp] OR Comoros[Mesh:noexp] OR Congo[Mesh:noexp] OR Costa Rica[Mesh:noexp] OR Cote d'Ivoire[Mesh:noexp] OR Croatia[Mesh:noexp] OR Cuba[Mesh:noexp]OR Cyprus[Mesh:noexp] OR Czechoslovakia[Mesh:noexp] OR Czech Republic[Mesh:noexp] OR Slovakia[Mesh:noexp] OR Djibouti[Mesh:noexp] OR "Democratic Republic of the Congo"[Mesh:noexp] OR Dominica[Mesh:noexp] OR Dominican Republic[Mesh:noexp] OR East Timor[Mesh:noexp]OR Ecuador[Mesh:noexp] OR Egypt[Mesh:noexp] OR El Salvador[Mesh:noexp] OR Eritrea[Mesh:noexp] OR Estonia[Mesh:noexp] OR Ethiopia[Mesh:noexp] OR Fiji[Mesh:noexp] OR Gabon[Mesh:noexp] OR Gambia[Mesh:noexp] OR "Georgia (Republic)"[Mesh:noexp] OR Ghana[Mesh:noexp]OR Greece[Mesh:noexp] OR Grenada[Mesh:noexp] OR Guatemala[Mesh:noexp] OR Guinea[Mesh:noexp] OR Guinea-Bissau[Mesh:noexp] OR Guam[Mesh:noexp] OR Guyana[Mesh:noexp] OR Haiti[Mesh:noexp] OR Honduras[Mesh:noexp] OR Hungary[Mesh:noexp] OR India[Mesh:noexp] OR Indonesia[Mesh:noexp]OR Iran[Mesh:noexp] OR Iraq[Mesh:noexp] OR Jamaica[Mesh:noexp] OR Jordan[Mesh:noexp] OR Kazakhstan[Mesh:noexp] OR Kenya[Mesh:noexp] OR Korea[Mesh:noexp] OR Kosovo[Mesh:noexp] OR Kyrgyzstan[Mesh:noexp] OR Laos[Mesh:noexp] OR Latvia[Mesh:noexp] OR Lebanon[Mesh:noexp]OR Lesotho[Mesh:noexp] OR Liberia[Mesh:noexp] OR Libya[Mesh:noexp] OR Lithuania[Mesh:noexp] OR Macedonia[Mesh:noexp] OR Madagascar[Mesh:noexp] OR Malaysia[Mesh:noexp] OR Malawi[Mesh:noexp] OR Mali[Mesh:noexp] OR Malta[Mesh:noexp] OR Mauritania[Mesh:noexp]OR Mauritius[Mesh:noexp] OR Mexico[Mesh:noexp] OR Micronesia[Mesh:noexp] OR Middle East[Mesh:noexp] OR Moldova[Mesh:noexp] OR Mongolia[Mesh:noexp] OR Montenegro[Mesh:noexp] OR Morocco[Mesh:noexp] OR Mozambique[Mesh:noexp] OR Myanmar[Mesh:noexp] OR Namibia[Mesh:noexp]OR Nepal[Mesh:noexp] OR Netherlands Antilles[Mesh:noexp] OR New Caledonia[Mesh:noexp] OR Nicaragua[Mesh:noexp] OR Niger[Mesh:noexp] OR Nigeria[Mesh:noexp] OR Oman[Mesh:noexp] OR Pakistan[Mesh:noexp] OR Palau[Mesh:noexp] OR Panama[Mesh:noexp] OR Papua New Guinea[Mesh:noexp]OR Paraguay[Mesh:noexp] OR Peru[Mesh:noexp] OR Philippines[Mesh:noexp] OR Poland[Mesh:noexp] OR Portugal[Mesh:noexp] OR Puerto Rico[Mesh:noexp] OR Romania[Mesh:noexp] OR Russia[Mesh:noexp] OR "Russia (Pre-1917)"[Mesh:noexp] OR Rwanda[Mesh:noexp] OR "Saint Kitts and Nevis"[Mesh:noexp] OR Saint Lucia[Mesh:noexp] OR "Saint Vincent and the Grenadines"[Mesh:noexp] OR Samoa[Mesh:noexp] OR Saudi Arabia[Mesh:noexp] OR Senegal[Mesh:noexp] OR Serbia[Mesh:noexp] OR Montenegro[Mesh:noexp] OR Seychelles[Mesh:noexp] OR Sierra Leone[Mesh:noexp] OR Slovenia[Mesh:noexp] OR Sri Lanka[Mesh:noexp] OR Somalia[Mesh:noexp] OR South Africa[Mesh:noexp] OR Sudan[Mesh:noexp] OR Suriname[Mesh:noexp] OR Swaziland[Mesh:noexp] OR Syria[Mesh:noexp] OR Tajikistan[Mesh:noexp] OR Tanzania[Mesh:noexp]OR Thailand[Mesh:noexp] OR Togo[Mesh:noexp] OR Tonga[Mesh:noexp] OR "Trinidad and Tobago"[Mesh:noexp] OR Tunisia[Mesh:noexp] OR Turkey[Mesh:noexp] OR Turkmenistan[Mesh:noexp] OR Uganda[Mesh:noexp] OR Ukraine[Mesh:noexp] OR Uruguay[Mesh:noexp] OR USSR[Mesh:noexp]OR Uzbekistan[Mesh:noexp] OR Vanuatu[Mesh:noexp] OR Venezuela[Mesh:noexp] OR Vietnam[Mesh:noexp] OR Yemen[Mesh:noexp] OR Yugoslavia[Mesh:noexp] OR Zambia[Mesh:noexp] OR Zimbabwe[Mesh:noexp])

For peer review only - http://bmjopen.bmj.com/site/about/guidelines.xhtml

Appendix 2. Extraction sheet

Appendix 2, Extraction terms	
**Background details**	First author surname<br>Year<br>Title<br>Country<br>Region<br>Intervention- Funding source<br>Evaluation- Funding source<br>Author email address
**Study Context**	Study location:<br>rural=1;<br>urban=2;<br>peri-urban=3;<br>mixed (urban/rural/peri-urban)=4<br>Background and history of groups<br>(who formed, trained and maintained the groups in the study areas)<br>Health system strength/indicators<br>(in author's words- the health systems setting, the important health indicators talked about in the study area)<br>Specific policy environment towards groups (NRLM, linkages with other devlopment programmes)<br>Others
**Hypothesis tested |&| Theory of Change**	What is the underlying hypothesis being tested in the paper?<br>What is the implicit or explicit theory of change?<br>Were any statistical analyses done to test the TOC?<br>Yes/ No- 1/0<br>What are the effect modifiers/ anticipated effect modifiers, if any<br>What SBC theory is used implicitly or explicitly?
**Intervention**	Describe the intervention and its components in the authors' own words<br>Intervention description- On field implementation details (Text from paper/ rewrite in brief)<br>What elements of the intervention were participatory (in authors' own words)<br>What is the average group size?<br>Who was the facilitator?<br>(1- Internal member, trained from within the group; 2- External facilitator, from NGO or Govt.)<br>What training was given to the facilitator in authors' own words?<br>Were the facilitators given any monetary incentive? Y=1, N=0<br>Number of groups per facilitator (con)<br>Number of groups per facilitator (exp)

	intervention period?<br>Number of sessions<br>Is this a mixed method evaluation? Yes=1, No=2
**Group details**	How often did groups meet?<br>Underlying group type:-<br>1= Microfinance groups<br>2= Open groups<br>3= Community based women only groups<br>4= Special population groups<br>What is the duration of each meeting?<br>Population coverage of groups? (% coverage)<br>What are the service linkages (health specific), if any?
**Intensity/ Coverage of Intervention**	Details on functioning of Groups in Control arm<br>Details on functioning of Groups in Intervention arm<br>Topics discussed in a regular (control) group meeting + length of discussion<br>Additional topics discussed in Intervention arm group meetings (Due to layering) + length of discussion<br>Exposure to the intervention (text from paper, if any)
**Participant Inclusion Criterion**	Evaluation-<br>Participant eligibility, inclusion criteria [text]<br>Evaluation-<br>Participant eligibility, Exclusion criteria [text]<br>Intervention-<br>Participant eligibility, inclusion criteria [text]<br>Intervention-<br>Participant eligibility, Exclusion criteria [text]
**Evaluation details**	How would you characterise the evaluation on the efficacy/effectiveness spectrum?<br>1 = towards efficacy (implementation on small/medium scale by non-gov partners);<br>2 = implementation on small/medium scale by gov partners in conditions close to routine;<br>3 = implementation on large scale, through routine training systems by gov partners<br>Evaluation design (1= RCT,<br>2=Longitudinal Cohort,<br>3= Repeated Cross-sectional survey<br>4= Pre/ post evaluation design<br>5=Other (specify, eg. Type of Quasi experimental)<br>Nature of comparison (1=exp vs con; 2=before vs after intervention)<br>Is there a control group? Yes=1, No=2<br>Type of control (group with no intervention OR no group, with or without matching)

	Is this a cross-sectional baseline/endline survey design? Yes=1, No=2<br>Please describe survey design [text]
**Cost effectiveness component**	Does the study include a cost effectiveness component?<br>Yes--1<br>No--2<br>Is the cost given a total cost figure, or separate cost given for each descriptive component of the intervention?<br>Total cost--1<br>Individual cost components--2<br>Cost effectiveness method used (text from paper)<br>Cost value (figure given in the paper with unit)
**Outcome areas**	MNCH<br>SRH and HIV<br>Family planning<br>General health/morbidity<br>Violence<br>Mental health<br>Nutrition<br>(Includes Breastfeeding)<br>Non-communicable diseases<br>Communicable diseases<br>Expenditure<br>Health insurance<br>Sanitation<br>Outcomes at group level or population level?<br>1- Group level<br>2- Population/ Community level<br>3. Sample members and non-members (from the community)<br>Non health outcomes, if any?<br>What is the primary outcome?<br>What are the secondary outcomes?<br>Does study differentiate between primary and secondary outcomes? Yes=1/no=0
**Timing of data collection**	baseline<br>endline<br>Time between baseline and endline<br>other information (if any, write it in text)
**Data collection and statistical methods**	Sampling methods in the authors' own words<br>Is the analysis by Intention-to-treat> (yes=1, no=0)<br>Confounders assessed/adjusted for?<br>Unit of clustering<br>Clustering at --<br>1: geographical level, 2: clusters formed using specific characteristics (other than geography)<br>Number and size of clusters<br>Adjusted for clustering in analysis? Yes=1/no=0

For peer review only - http://bmjopen.bmj.com/site/about/guidelines.xhtml

	Does the study have a sample size calculation? Yes=1, No=0<br>Does the study have a published protocol or registration with details of outcomes and analysis methods? Yes=1, No=0<br>Randomisation method, if any<br>Blinding process, in authors' own words (i.e. were any or all of persons who did random allocation, data collectors, and analysts blind to exposure/cont rolallocation?).<br>N eligible participants (exp vs control or before/after)<br>Method of assessment (write name of questionnaire/describe method)<br>e.g specific scales used like PHQ, CESD etc or anthropometric or biometric measurements taken<br>Statistical method (the name of analysis)
**Results**	mean age- separate baseline and endline if appropriate<br>Literacy - separate baseline and endline if appropriate<br>% of participants belonging to marginalised groups (con) - separate baseline and endline if appropriate
	N eligible participants (exp vs control or before/after)
**Results: Primary Outcomes**	Results - Mean (SD) in exp/con or before/after participants<br>Results - Rate in exp/con or before/after participants<br>Results - Prevalence in exp/con or before/after participants<br>Results - incidence in exp/con or before/after participants<br>Results -Odds Ratio (95% CI) for exp/con or before/after participants specify adjusted and unadjusted<br>Results - Rate ratio (95% CI) for exp/con or before/after participants specify adjusted and unadjusted<br>Results; Beta coefficient (95% CI) for exp/con or before/after participants specify adjusted and unadjusted<br>Results: difference-in-difference (DID) (difference in % points)- control v/s intervention before vs after
	Results; P-value for difference between groups
**Results: Secondary Outcomes**	Results: report all results on secondary outcomes with effect sizes (95% CI) and p-value - use ITT results, not sub-group analyses - Highlight significant findings (CI does not include 1) in red or with asterisk<br>Notes on secondary outcomes
**Qualitative Results and Appraisal**	Qualitative sample<br>Qualitative data collection methods<br>Qualitative results<br>Clarity of the research question<br>Explanation of the context<br>Sampling<br>Reflexivity of the account<br>Clear account of the methods used for data collection and analysis

	Amount of data pertinent to the review question
**Authors' Own Account Of Study Limitations**	Notes on bias
**Risk of Bias assessment**	ROB-2 and ROBINS
**Barriers And Enablers To Intervention Achieving Impact**	Enablers for the intervention in the authors words<br>Barriers for the intervention in the authors words<br>Reviewers interpretation of barriers and enablers.
**Layering specific Barriers and Enablers**	Barriers to layering the intervention<br>Enablers to layering the intervention
**Process Evaluation**	Does this study include a process evaluation<br>Is the process evaluation quantitative, qualitative or mixed methods (give citation here)
**Additional information, if any**	Notes/ Remarks

For peer review only

For peer review only

For peer review only - http://bmjopen.bmj.com/site/about/guidelines.xhtml

PRISMA-P (Preferred Reporting Items for Systematic review and Meta-Analysis Protocols) 2015 checklist: recommended items to address in a systematic review protocol*

Section and topic	Item No	Checklist item	Page number
**ADMINISTRATIVE INFORMATION**			
Identification	1a	Identify the report as a protocol of a systematic review	Title, p. 1; abstract, p. 2; Introduction, p. 4
Update	1b	If the protocol is for an update of a previous systematic review, identify as such	Not an update
Registration	2	If registered, provide the name of the registry (such as PROSPERO) and registration number	Abstract, p. 3, PROSPERO registration number provided
Authors:			
Contact	3a	Provide name, institutional affiliation, e-mail address of all protocol authors; provide physical mailing address of corresponding author	Title page, p. 1
Contributions	3b	Describe contributions of protocol authors and identify the guarantor of the review	Author contributions, p. 13
Amendments	4	If the protocol represents an amendment of a previously completed or published protocol, identify as such and list changes; otherwise, state plan for documenting important protocol amendments	Plans to report protocol amendments noted in Ethics and Dissemination, p. 11
Support:			
Sources	5a	Indicate sources of financial or other support for the review	Funding statement, p. 13
Sponsor	5b	Provide name for the review funder and/or sponsor	
Role of sponsor or funder	5c	Describe roles of funder(s), sponsor(s), and/or institution(s), if any, in developing the protocol	Funding statement, p. 13
INTRODUCTION			
Describe the rationale for the review in the context of what is already known			Introduction, pp. 4-5
Objectives	7	Provide an explicit statement of the question(s) the review will address with reference to participants, interventions, comparators, and outcomes (PICO)	Introduction, p. 5
METHODS			
Eligibility criteria: Specify the study characteristics (such as PICO, study design, setting, time frame) and report characteristics (such as years considered, language, publication status) to be used as criteria for eligibility for the review			Study design, Participants, Types of Interventions, pp. 5-7

For peer review only

Information sources	9	Describe all intended information sources (such as electronic databases, contact with study authors, trial registers or other grey literature sources) with planned dates of coverage	Information sources, p. 8
Search strategy	10	Present draft of search strategy to be used for at least one electronic database, including planned limits, such that it could be repeated	Search strategy, p. 8; Appendix 1
Study records:			
Data management	11a	Describe the mechanism(s) that will be used to manage records and data throughout the review	Data management, pp. 8-9
Selection process	11b	State the process that will be used for selecting studies (such as two independent reviewers) through each phase of the review (that is, screening, eligibility and inclusion in meta-analysis)	Selection strategy, p. 9
Data collection process	11c	Describe planned method of extracting data from reports (such as piloting forms, done independently, in duplicate), any processes for obtaining and confirming data from investigators	Data collection process, p. 9
Data items	12	List and define all variables for which data will be sought (such as PICO items, funding sources), any pre-planned data assumptions and simplifications	Data items, pp. 9-10; Appendix 2
Outcomes and prioritization	13	List and define all outcomes for which data will be sought, including prioritization of main and additional outcomes, with rationale	Outcomes, pp. 7-8; Outcomes and prioritisation, p. 10
Risk of bias in individual studies	14	Describe anticipated methods for assessing risk of bias of individual studies, including whether this will be done at the outcome or study level, or both; state how this information will be used in data synthesis	Risk of bias in individual studies, p. 10
Data synthesis	15a	Describe criteria under which study data will be quantitatively synthesised	Data synthesis, pp. 10-11
Data synthesis Meta-bias(es)	15b	If data are appropriate for quantitative synthesis, describe planned summary measures, methods of handling data and methods of combining data from studies, including any planned exploration of consistency (such as I^2, Kendall's τ)	Data synthesis, pp. 10-11
	15c	Describe any proposed additional analyses (such as sensitivity or subgroup analyses, meta-regression)	N/A
	15d	If quantitative synthesis is not appropriate, describe the type of summary planned	Data synthesis, pp. 10-11
	16	Specify any planned assessment of meta-bias(es) (such as publication bias across studies, selective reporting within studies)	Meta-biases, p. 11
Confidence in cumulative evidence	17	Describe how the strength of the body of evidence will be assessed (such as GRADE)	Confidence in cumulative evidence, p. 11

For peer review only

* It is strongly recommended that this checklist be read in conjunction with the PRISMA-P Explanation and Elaboration (cite when available) for important clarification on the items. Amendments to a review protocol should be tracked and dated. The copyright for PRISMA-P (including checklist) is held by the PRISMA-P Group and is distributed under a Creative Commons Attribution Licence 4.0.

From: Shamseer L, Moher D, Clarke M, Ghersi D, Liberati A, Petticrew M, Shekelle P, Stewart L, PRISMA-P Group. Preferred reporting items for systematic review and meta-analysis protocols (PRISMA-P) 2015: elaboration and explanation. BMJ. 2015 Jan 2;349(jan02 1):g7647.

For peer review only

BMJ Open

Effects of integrated economic and health interventions with women's groups on health-related knowledge, behaviours and outcomes in low-and middle-income countries: a systematic review protocol

Journal:	*BMJ Open*
Manuscript ID	bmjopen-2020-046856.R1
Article Type:	Protocol
Date Submitted by the Author:	23-Mar-2021
Complete List of Authors:	Desai, Sapna; Population Council India<br>Mehta, Kala; University of California San Francisco<br>Singh, Roopal; Population Council India<br>Westley, Allie; Stanford University<br>Dirisu, Osasuyi; Population Council Nigeria<br>Wong, Connie; Lane Medical Library; Stanford University School of Medicine<br>De Hoop, Thomas; American Institutes for Research<br>Darmstadt, Gary; Stanford University School of Medicine, Pediatrics
**Primary Subject Heading**:	Public health
Secondary Subject Heading:	Global health, Public health
Keywords:	PUBLIC HEALTH, HEALTH ECONOMICS, International health services < HEALTH SERVICES ADMINISTRATION & MANAGEMENT, Health economics < HEALTH SERVICES ADMINISTRATION & MANAGEMENT

SCHOLARONE™
Manuscripts

I, the Submitting Author has the right to grant and does grant on behalf of all authors of the Work (as defined in the below author licence), an exclusive licence and/or a non-exclusive licence for contributions from authors who are: i) UK Crown employees; ii) where BMJ has agreed a CC-BY licence shall apply, and/or iii) in accordance with the terms applicable for US Federal Government officers or employees acting as part of their official duties; on a worldwide, perpetual, irrevocable, royalty-free basis to BMJ Publishing Group Ltd ("BMJ") its licensees and where the relevant Journal is co-owned by BMJ to the co-owners of the Journal, to publish the Work in this journal and any other BMJ products and to exploit all rights, as set out in our licence.

The Submitting Author accepts and understands that any supply made under these terms is made by BMJ to the Submitting Author unless you are acting as an employee on behalf of your employer or a postgraduate student of an affiliated institution which is paying any applicable article publishing charge ("APC") for Open Access articles. Where the Submitting Author wishes to make the Work available on an Open Access basis (and intends to pay the relevant APC), the terms of reuse of such Open Access shall be governed by a Creative Commons licence – details of these licences and which Creative Commons licence will apply to this Work are set out in our licence referred to above.

Other than as permitted in any relevant BMJ Author's Self Archiving Policies, I confirm this Work has not been accepted for publication elsewhere, is not being considered for publication elsewhere and does not duplicate material already published. I confirm all authors consent to publication of this Work and authorise the granting of this licence.

For peer review only

Effects of integrated economic and health interventions with women's groups on health-related knowledge, behaviours and outcomes in low-and middle-income countries: a systematic review protocol

Short title: Effects of women's groups with integrated economic and health interventions

Sapna Desai,[1] Kala M. Mehta,[2] Roopal Jyoti Singh,[1] Allie K. Westley, [3] Osasuyi Dirisu, [4] Connie Wong, [5] Thomas de Hoop,[6] Gary L. Darmstadt[7]

[1]Population Council, New Delhi, India, sdesai@popcouncil.org, ORCID #

[2]University of California, San Francisco, CA, USA, kmehta23@stanford.edu

[3] Stanford University, Stanford, CA, USA, awestley@stanford.edu

[4] Population Council, Abuja, Nigeria, odirisu@popcouncil.org

[5]Lane Medical Library, Stanford University School of Medicine, Stanford, CA, USA, hongnei@stanford.edu

[6]American Institutes for Research, Washington DC, USA, tdehoop@air.org

[7]Department of Pediatrics, Stanford University School of Medicine, Stanford, CA, USA, gdarmsta@stanford.edu

Corresponding author: Gary L. Darmstadt, MD, MS, Department of Pediatrics, Stanford University School of Medicine, 1701 Page Mill Road, Palo Alto, CA 94304, USA, gdarmsta@stanford.edu

Word Count: Abstract, 251, body, 2502.

For peer review only

Abstract

Introduction

Economic groups such as microfinance or self-help groups (SHGs) are widely implemented in low and middle-income countries (LMICs). Women's groups are voluntary groups which aim to improve members' well-being through activities such as joint savings, credit, livelihoods development and/or health activities. Health interventions are increasingly added on to existing women's economic groups as a public health intervention for women and their families. Here we present the protocol for a mixed-methods systematic review we will conduct of the evidence on integrated economic and health interventions upon women's groups to assess whether and how they improve health-related knowledge, behaviour and outcomes in LMICs.

Methods and analysis

We will search seven electronic databases for published literature, along with manual searches and consultation. The review will include (i) randomised trials and non-randomised quasi-experimental studies of intervention effects of integrated economic and health interventions delivered through women's groups in LMICs, and (ii) sibling studies that examine factors related to intervention content, context, implementation processes and costs. We will appraise risk of bias and study quality using standard tools. High and moderate-quality studies will be grouped by health domain and synthesised without meta-analysis. Qualitative evidence will be thematically synthesised and integrated into the quantitative synthesis using a matrix approach.

Ethics and dissemination

This protocol was reviewed and deemed exempt by the institutional review board at the American Institutes for Research. Findings will be shared through peer-reviewed publication and disseminated with programme implementers and policymakers engaged with women's groups.

For peer review only

For peer review only - http://bmjopen.bmj.com/site/about/guidelines.xhtml

Registration

This protocol is registered as PROSPERO CRD42020199998.

Strengths and limitations of this study

- We outline the protocol for a systematic review of current global literature exploring a highly important method of health education delivery for women.
- This convergent mixed-methods review will utilise robust methods to integrate quantitative evidence on intervention effectiveness with qualitative studies to provide programme and policy-relevant evidence on the effects of integrated women's groups interventions on health outcomes.
- The review will include sibling studies such as process evaluations identified through a comprehensive search of published literature and will summarise intervention content, context, implementation processes, facilitating factors and barriers, and costs.
- The review will include a comprehensive risk-of-bias assessment to critically appraise study quality.
- The review is limited by lack of meta-analysis, given heterogeneity of reported outcomes.

For peer review only

For peer review only - http://bmjopen.bmj.com/site/about/guidelines.xhtml

Introduction

Women's groups come together in many settings in low and middle-income countries (LMICs) to improve women's opportunities for livelihoods and financial security.[1] Women's self-help groups (SHGs) and collectives have developed and spread throughout India for several decades; the National Rural Livelihoods Mission (NRLM) has reached over 70 million households with microfinance and livelihoods interventions through SHGs since 2011.[2] Similarly, Village Savings and Loan Associations (VSLA) and other savings groups have operated for in many parts of Africa for over three decades.[3] Economic women's groups typically focus on savings, access to credit and livelihoods to improve financial inclusion and economic outcomes. A recent systematic review found that livelihoods groups have small but positive effects on consumption and moderate effects on savings, but did not find effects on income, asset ownership or labour force participation.[1] The review suggests that livelihoods groups may have more impact when combined with investments in human capital, including health. Previous reports of the effect of combined microfinance and health interventions, though not all group-based, suggest some evidence of improvements in health behaviours amongst participants.[4 5]

Governments and other stakeholders increasingly support integrating (or "layering") additional activities onto livelihoods-based groups, most commonly with health interventions. Combined economic and health interventions implemented through groups have been evaluated for impacts on a range of health outcomes including violence against women,[6 7] health-promoting behaviours[8] and malnutrition amongst women and children.[9] Despite growing policy interest, there has been no systematic review of the evidence on combined economic and health interventions through women's groups to inform ongoing and future programmes in low and middle-income settings. We build on previous reviews by (i) focusing on the combined effect of group-based economic and health programs, as distinct from previous reviews that examined the health impacts of joining a microfinance-based group with or without layering[4]

For peer review only - http://bmjopen.bmj.com/site/about/guidelines.xhtml

or non-group-based microfinance interventions[5] and (ii) including studies across all LMICs rather than a specific region [10 11].

This paper presents the protocol, following Preferred Reporting Items for Systematic Review and Meta-analysis Protocols (PRISMA-P) guidelines,[10] for a review we will conduct which aims to address this gap through synthesising quantitative and qualitative evidence on the health effects of combined group-based economic and health interventions and implementation factors that contributed to achieving intended outcomes. We aim to answer two primary questions: 1) What is the effect of combined economic and health, nutrition and/or sanitation interventions delivered through women's groups on health outcomes amongst women and children in LMICs, compared to single-purpose, non-layered (i.e., only economic or only health) interventions with women's groups or to no group intervention, and 2) What factors related to intervention content, context and implementation processes are enablers or barriers to achieving health outcomes?

Methods

Study design

Study inclusion criteria

This review is a mixed-methods convergent review in which synthesis of quantitative effects and qualitative evidence for factors in intervention implementation will be combined within the review.[12] Our review will include all trials – both randomised controlled trials and non-randomised, quasi-experimental studies – on integrated economic and health interventions through groups. Non-randomised studies of interventions (NRSI) include stronger and weaker designs.[13] Criteria for inclusion of NRSI will be ability to address selection bias either through the inclusion of a baseline measurement of the outcome of interest or other relevant confounding factors, or through intervention allocation rules that enable the use of regression discontinuity designs or natural

For peer review only - http://bmjopen.bmj.com/site/about/guidelines.xhtml

experiments.[14 15] Additional inclusion criteria include that the intervention was implemented in a LMIC according to World Bank classification[16] and the study was published between January 1, 2000 and May 31, 2020 in the peer-reviewed or grey literature. We will also include studies which are linked to the included randomised and non-randomised trials, including process evaluations, qualitative studies and cost or cost-effectiveness studies, i.e. "trial sibling studies."[17]

Study exclusion criteria

Studies with the following characteristics will be excluded: 1) No qualitative or quantitative data were collected to evaluate the effect of an intervention, for example the study only provides observational descriptions of groups; 2) The treatment group only implements an economic or health intervention, i.e. the intervention is not an integrated approach; 3) No comparison group or baseline measurement was included, 4) An independent study such as a process evaluation, qualitative study and cost or cost-effectiveness study on women's groups was not linked to a randomised or non-randomised impact evaluation (non-sibling studies); and 5) The study was published prior to January 1, 2000.

Participants

Participant inclusion criteria

For this review, we define an eligible woman's group as a group that was constituted for women to regularly exchange information, support or goods (e.g. savings) and plan individual and/or collective action. Mixed-sex groups will be included if there were 51% or more women or authors note the group was composed mostly of women. We will include closed groups and groups constituted for women that were also open to others to attend meetings. We define adult women to be 18 years of age or more.

Participant exclusion criteria

For peer review only - http://bmjopen.bmj.com/site/about/guidelines.xhtml

Our review will exclude men's only groups, family groups, select committees of community representatives, professional groups and political associations. We will also exclude adolescent girls' groups, as a recent systematic review examines these groups and the role of integrated interventions compared to singular approaches,[18] as well as groups that did not have a joint purpose or objective to meet regularly, such as short-term training sessions.

Types of interventions

Intervention inclusion criteria

The review will focus on integrated economic and health/sanitation/nutrition interventions delivered through women's' groups. Groups may be pre-existing in the study areas or may have been set up as part of the group intervention. Interventions will include combinations of health and/or nutrition and/or sanitation components with an economic intervention aimed to improve financial inclusion, income, consumption or livelihoods, such as microfinance, entrepreneurial skills training or collective enterprise development.

Intervention exclusion criteria

We will not examine economic women's groups that did not have a health, sanitation or nutrition intervention component (defined below), or women's groups that solely had health interventions, without economic interventions, such as mothers' groups. If the intervention was not described in sufficient detail to categorise the type of intervention, it will not be included.

Comparisons

Comparisons will include groups with non-intervention controls and 'usual' practice. We will include evaluations that compared the integrated intervention to either no group or to a singular model (economic or health). For example, groups with an economic component but no health, sanitation, or nutrition intervention can serve as the comparison group.

For peer review only - http://bmjopen.bmj.com/site/about/guidelines.xhtml

Outcomes

Condition or domain being studied will include any health, nutrition or sanitation-related knowledge, behaviours and outcomes across all domains consistent with the World Health Organisation definition of health as 'a state of complete physical, mental and social wellbeing.'[19] Domains will include (but are not limited to): maternal, newborn and child health; sexual and reproductive health; nutrition; infectious and vector-borne disease; non-communicable disease; mental health; violence against women; water, sanitation and hygiene; health services utilisation; and health expenditure. The domain of maternal health, for example, may include both objectively measured health outcomes such as maternal mortality and anaemia, along with behaviours during the perinatal period and knowledge of postnatal care. Infectious and vector-borne disease may include outcomes related to vector density or incidence of malaria, along with behaviours such as sleeping under a bednet or seeking treatment for fever and knowledge of symptoms of malaria or dengue.

Information sources

Electronic databases

We will search the following seven electronic research databases: Pubmed, Scopus, Embase, Web of Science, PsycINFO, Social Sciences Citation Index (SSCI), and EconLit.

Other sources

Studies will also be obtained for the same time period from reference lists of included studies, reference lists of systematic reviews on the effects of women's groups, the 3ie Database of Impact Evaluations and Evidence Consortium on Women's Groups repository, and Google Scholar. In addition, we will contact study authors to identify process evaluations or qualitative studies conducted in conjunction with an impact evaluation and consult with experts familiar with

For peer review only

For peer review only - http://bmjopen.bmj.com/site/about/guidelines.xhtml

interventions in Francophone Africa and Latin/South America to recommend inclusion of French or Spanish language articles. We will also contact study authors of published process evaluations or qualitative studies to identify linked, unpublished impact evaluations. Lastly, we will consult with experts in this area, both researchers and practitioners, to review our list of included studies to identify any interventions that may have been excluded.

Search strategy

The search strategy for PubMed is in Appendix 1. This strategy will be adapted to the other electronic databases by the library scientist on our team (CW), with any modifications reported in the review manuscript.

Data management

The titles and abstracts retrieved by electronic searches will be exported to a systematic review application called COVIDENCE. Reference management for full text will be conducted using Endnote.

Selection strategy

Two reviewers will independently screen abstracts and titles. The reviewers will not be blind to the author or journal information. The abstracts of papers that are in a language other than English will be translated using Google Translate. If considered eligible or eligibility is unclear, professional translation of the full paper will be undertaken. The full texts of articles will be obtained for all potentially eligible studies for further examination. For all excluded articles, the primary reason for exclusion will be recorded and documented in the excluded studies table. Discrepancies between the two review authors regarding study eligibility will be resolved by discussion and consensus and if necessary, by a third reviewer.

For peer review only - http://bmjopen.bmj.com/site/about/guidelines.xhtml

Data collection process

Two investigators will independently collect data from articles selected for full text review using an extraction form developed by the authors and piloted before use. Discrepancies between reviewers regarding data extraction will be resolved by discussion and consensus, and if necessary, by contacting authors.

Data items

The following information will be extracted into COVIDENCE and an Excel file (see the full list of data extraction items in Appendix 2): Publication details: authors, year and journal; study context and study design; hypothesis tested and theory of change; intervention design; level of community participation; social and behaviour change approach; participant inclusion criteria and demographic characteristics; characteristics of the intervention, including the duration, intervention strategies, intensity and coverage; outcomes (primary and secondary); data collection and statistical methods, including sample size; findings of process evaluations and cost effectiveness; source(s) of research funding and potential conflicts of interest; and study limitations in the authors' own words. Attempts will be made to contact the corresponding authors of included studies if there are specific queries regarding the items for extractions that are unavailable in the published manuscript.

Outcomes and prioritisation

We will extract data on all health outcomes, as detailed above. Primary outcomes, as identified by the authors, will be prioritised in reporting. If authors have not distinguished between primary and secondary outcomes, we will report all outcomes. In addition, we will extract data on non-health outcomes reported by authors, such as on women's empowerment or economic outcomes.

Risk of bias in individual studies

For peer review only - http://bmjopen.bmj.com/site/about/guidelines.xhtml

We will appraise risk of bias in trials using the Cochrane guides for randomised controlled trials (Cochrane ROB-2) and for assessing risk of bias in non-randomised studies of interventions (ROBINS-1).[20, 21] We will examine risk of bias for primary outcomes. If a primary outcome is not specified, we will utilise the outcome for which sample size was determined. Process evaluations will be appraised using an adapted version of an eight-item tool developed by the EPPI Centre, with additions specific to group-based interventions.[22 23]

Data synthesis

We will first summarise characteristics of all trials with their risk of bias appraisal, grouped by health domain. Given the anticipated heterogeneity of health outcomes, we do not plan to conduct a meta-analysis. We will synthesise findings from trials with low or moderate risk of bias in tabular form and in the narrative text. We will present forest plots by health domain without a summary measure if possible, and potentially present harvest plots to compare effects by factors related to the health intervention approach. Synthesis methods and results will be presented according to the Synthesis without meta-analysis (SWiM) guidelines.[24]

Findings from process evaluations and other qualitative studies will be analysed separately in a thematic synthesis.[25] We will integrate the synthesis on intervention effects with the qualitative synthesis by juxtaposing findings in a matrix.[26] If consistent programme theories emerge across interventions, we may conduct a synthesis by programme theory.

Meta-biases

We will plot study sample size against effect size to evaluate whether these are skewed and asymmetrical in the presence of publication bias and other biases.[27] In addition, the risk of bias assessment tools will identify whether studies indicate selective reporting of outcomes, which we will summarise across studies if possible.

For peer review only - http://bmjopen.bmj.com/site/about/guidelines.xhtml

Confidence in cumulative evidence

The overall quality of evidence on outcomes will be presented using the Grades of Recommendation, Assessment, Development and Evaluation (GRADE) criteria.[28] This method will include an evaluation of within-study risk of bias (methodological quality), directness of evidence, heterogeneity, precision of effect estimates and risk of publication bias.

Patient and public involvement

There will be no patient or public involvement in the conduct or dissemination of the results of this study.

Ethics and dissemination

Our findings will be submitted for peer-reviewed publication. Deviations from the study protocol will be noted in the manuscript. Findings will be disseminated through conference presentations and shared with both programme implementers and policymakers engaged in interventions with women's groups, as well as on the website of the Evidence Consortium on Women's Groups (www.womensgroupevidence.org). No primary data collection will be undertaken and the institutional review board at the American Institutes for Research in Washington, D.C, determined that the review was exempt from human subjects review.

Discussion

This proposed review will contribute to a growing body of literature regarding how different types of women's groups may contribute to improved health outcomes.[4 10 11] The focus on integrated, group-based interventions is novel and also timely, as several countries and organisations are considering scaling these interventions in a variety of geographic contexts. Building a

For peer review only

systematically aggregated, critically appraised knowledge base will aid in discerning the up-to-date evidence base regarding the health effects of integrated economic and health interventions. This review will also report on different types of women's groups, building on existing typologies,[29] to advance understanding on how interventions may be integrated. Systematically synthesising the evidence for health effects as well as facilitating factors in creating health benefits will aid understanding of which integrated women's groups interventions will potentially work, for whom, in what context, and with which pre-conditions. Further, the incorporation of a mixed-methods approach and qualitative synthesis of implementation evidence will enable better understanding of how to implement women's group interventions effectively in various contexts.[26]

It is the intention that this review will be published in a peer-reviewed journal, will be presented at relevant global health and development conferences and the learnings will be disseminated to key-stakeholders working with or funding work with women's groups in LMIC contexts. Not only will this work be important in and of itself, it will also shape and define several new research questions that will be critical to the field as it moves forward.

Authors' contributions

SD, GLD and TdH conceputalised the review. SD and KM drafted the protocol, with critical inputs from all authors. CW developed the search methodology. RJS, OD and AW developed and piloted extraction sheets. All authors reviewed and approved the final protocol.

Funding statement

This work was supported by the Bill & Melinda Gates Foundation in support of the Evidence Consortium on Women's Groups, through grant number OPP1201417. The funder provided input to the protocol, but the senior author had responsibility for the content and the decision to publish the protocol.

For peer review only

Competing interest statement

The authors have no competing interests. If studies conducted by any author are included in the review, this will be noted in the manuscript for publication

For peer review only

For peer review only - http://bmjopen.bmj.com/site/about/guidelines.xhtml

References

1. Barooah B, Chinoy S, Dubey P, et al. Improving and sustaining livelihoods through group-based interventions: Mapping the evidence (3ie Evidence Gap Map Report 13). *New Delhi, India: International Initiative for Impact Evaluation (3ie)* 2019
2. NRLM. Deendayal Antyodaya Yojana - National Rural Livelihoods Mission 2011 [Available from: https://aajeevika.gov.in/en accessed 15.9.2020.
3. Hendricks L, Chidiac S. Village savings and loans: A pathway to financial inclusion for Africa's poorest households. *Enterprise Development and Microfinance* 2011;22(2):134-46.
4. Orton L, Pennington A, Nayak S, et al. Group-based microfinance for collective empowerment: a systematic review of health impacts. *Bulletin of the world health organization* 2016;94(9):694.
5. Lorenzetti LM, Leatherman S, Flax VL. Evaluating the effect of integrated microfinance and health interventions: an updated review of the evidence. *Health policy and planning* 2017;32(5):732-56.
6. Pronyk PM, Hargreaves JR, Kim JC, et al. Effect of a structural intervention for the prevention of intimate-partner violence and HIV in rural South Africa: a cluster randomised trial. *The lancet* 2006;368(9551):1973-83.
7. Jejeebhoy SJ, Santhya K, Acharya R, et al. Empowering women and addressing violence against them through self-help groups (SHGs): Population Council New Delhi, India 2017.
8. Flax VL, Negerie M, Ibrahim AU, et al. Integrating group counseling, cell phone messaging, and participant-generated songs and dramas into a microcredit program increases Nigerian women's adherence to international breastfeeding recommendations. *The Journal of nutrition* 2014;144(7):1120-24.
9. Raghunathan K, Kumar N, Gupta S, et al. Learning together: Experimental evidence on the impact of group-based nutrition interventions in rural Bihar: Intl Food Policy Res Inst 2020.
10. Desai S, Misra M, Das A, et al. Community interventions with women's groups to improve women's and children's health in India: a mixed-methods systematic review of effects, enablers and barriers. BMJ Global Health 2020.
11. Kumar N, Scott S, Menon P, et al. Pathways from women's group-based programs to nutrition change in South Asia: A conceptual framework and literature review. *Global food security* 2018;17:172-85.
12. Noyes J, Booth A, Cargo M, et al. Qualitative evidence. *Cochrane Handbook for Systematic Reviews of Interventions* 2019:525-45.
13. Reeves BC, Wells GA, Waddington H. Quasi-experimental study designs series—paper 5: a checklist for classifying studies evaluating the effects on health interventions—a taxonomy without labels. *Journal of clinical epidemiology* 2017;89:30-42.
14. Cook TD, Wong VC. Better quasi-experimental practice. *The handbook of social research* 2008:134-65.
15. Shadish WR. Randomized controlled studies and alternative designs in outcome studies: Challenges and opportunities. *Research on Social Work Practice* 2011;21(6):636-43.
16. WorldBank. World Bank Country and Lending Groups 2020 [Available from: https://datahelpdesk.worldbank.org/knowledgebase/articles/906519-world-bank-country-and-lending-groups.
17. Moore GF, Audrey S, Barker M, et al. Process evaluation of complex interventions: Medical Research Council guidance. *bmj* 2015;350
18. Haberland NA, McCarthy KJ, Brady M. A systematic review of adolescent girl program implementation in low-and middle-income countries: Evidence gaps and insights. *Journal of Adolescent Health* 2018;63(1):18-31.

For peer review only - http://bmjopen.bmj.com/site/about/guidelines.xhtml

19. Organization WH. Preamble to the Constitution of WHO as adopted by the International Health Conference, 1946, 1946:100.
20. Higgins JP, Thomas J, Chandler J, et al. Cochrane handbook for systematic reviews of interventions: John Wiley & Sons 2019.
21. Sterne JA, Hernan MA, Reeves BC, et al. ROBINS-I: a tool for assessing risk of bias in non-randomised studies of interventions. *BMJ* 2016;355:i4919. doi: 10.1136/bmj.i4919 [published Online First: 2016/10/14]
22. Cargo M, Harris J, Pantoja T, et al. Cochrane Qualitative and Implementation Methods Group guidance series—paper 4: methods for assessing evidence on intervention implementation. *Journal of clinical epidemiology* 2018;97:59-69.
23. Rees R, Oliver K, Woodman J, et al. Children's views about obesity, body size, shape and weight: a systematic review. *EPPI-Centre, Institute of Education, University of London* 2009
24. Campbell M, McKenzie JE, Sowden A, et al. Synthesis without meta-analysis (SWiM) in systematic reviews: reporting guideline. *bmj* 2020;368
25. Thomas J, Harden A. Methods for the thematic synthesis of qualitative research in systematic reviews. *BMC medical research methodology* 2008;8(1):45.
26. Harden A, Thomas J, Cargo M, et al. Cochrane Qualitative and Implementation Methods Group guidance series—paper 5: methods for integrating qualitative and implementation evidence within intervention effectiveness reviews. *Journal of clinical epidemiology* 2018;97:70-78.
27. Sterne JA, Egger M. Funnel plots for detecting bias in meta-analysis: guidelines on choice of axis. *Journal of clinical epidemiology* 2001;54(10):1046-55.
28. Schünemann HJ, Higgins JP, Vist GE, et al. Completing 'Summary of findings' tables and grading the certainty of the evidence. *Cochrane Handbook for Systematic Reviews of Interventions* 2019:375-402.
29. Gram L, Desai S, Prost A. Classroom, club or collective? Three types of community- based group intervention and why they matter for health. *BMJ Glob Health (Forthcoming)* 2020

For peer review only

For peer review only - http://bmjopen.bmj.com/site/about/guidelines.xhtml

Effects of integrated economic and health interventions with women's groups on health-related knowledge, behaviours and outcomes in low-and middle-income countries: a systematic review protocol

Sapna Desai, Kala M. Mehta, Roopal Jyoti Singh, Allie K. Westley, Osasuyi Dirisu, Connie Wong, Thomas de Hoop, Gary L. Darmstadt

Supplementary File

Appendix 1. Electronic database search strategy

Appendix 1. Search strategy using PubMed

(Microfinance*[tw] OR microcredit*[TW] OR "micro-finance" OR "micro-credit" OR microloan* OR "micro-loan" OR microlending OR microinsurance OR "micro-insurance" OR "village bank" OR "savings group" OR "village banks" OR "savings groups" OR "self help group" OR "self help groups" OR "self-help group" OR "self-help groups" OR "self-help groups"[Mesh] OR "community mobilization"[TW]OR "community mobilization"[TW] OR "social mobilisation" OR "social mobilization" OR "community mobilisation"[TW] OR "financial support"[Mesh]) AND ("female"[Mesh] OR women OR "women"[Mesh] OR "mothers"[Mesh] OR "women's group" OR "women's groups" OR "women's group-based") AND (((health[TW] OR "health"[Mesh] OR "infections"[Mesh] OR infection[tw] OR infectious[tw] OR "sanitation"[Mesh] OR "waste management"[Mesh] OR toilet*[tw] OR "toilet facilities"[Mesh] OR "psychiatry"[Mesh] OR "mental health"[Mesh]OR wellbeing OR "well-being" OR "HIV infections"[Mesh] OR "HIV"[TW] OR "AIDS"[tw] OR "breastfeeding" OR "breast feeding"[Mesh] OR "breast feeding" OR "acquired immunodeficiency syndrome" OR "malaria" OR violen*[tw] OR "maternal health" OR "neonatal health" OR "child health" OR "family health" OR "infant health"[Mesh] OR "sexual health" OR "women's health"[Mesh] OR "maternal health"[Mesh] OR "newborn health" OR "safety" OR "family planning" OR immunization OR "vaccination"[Mesh] OR nutrition OR feeding[tiab] OR "malnutrition"[Mesh] OR "medication adherence"[Mesh] OR "health behaviors" OR "health behavior"[Mesh] OR "health behaviours" OR "health behaviour"[Mesh] OR "child growth"[Mesh] OR "water-borne"[tw] OR "vector-borne"[tw] OR "health awareness" OR "sexually transmitted diseases"[Mesh] OR "STD"[tiab] OR "sexually transmitted diseases"[tw]OR "school health" OR "Delivery, Obstetric"[Mesh]) AND (intervention*[tiab] OR program*[tiab] OR promotion[tiab] OR train*[tiab] OR education[TW] OR campaign*[TW] OR service*[Tiab] OR control*[tiab])) OR "health promotion"[Mesh] OR "health education"[Mesh]OR "health knowledge, attitudes, practice"[Mesh]) AND ("developing country"[tw] OR "developing countries"[tw] OR "developing nation"[tw] OR "developing nations"[tw] OR "developing population"[tw] OR "developing populations"[tw] OR "developing world"[tw] OR "less developed country"[tw] OR "less developed countries"[tw]OR "less developed nation"[tw] OR "less developed nations"[tw] OR "less developed population"[tw] OR "less developed populations"[tw] OR "less developed world"[tw] OR "lesser developed country"[tw] OR "lesser developed countries"[tw] OR "lesser developed nation"[tw]OR "lesser developed nations"[tw] OR "lesser developed population"[tw] OR "lesser developed populations"[tw] OR "lesser developed world"[tw] OR "under developed country"[tw] OR "under developed countries"[tw] OR "under developed nation"[tw] OR "under developed nations"[tw] OR "under developed population"[tw] OR "under developed populations"[tw] OR "under developed world"[tw] OR "underdeveloped country"[tw] OR "underdeveloped countries"[tw] OR "underdeveloped nation"[tw] OR "underdeveloped nations"[tw] OR "underdeveloped population"[tw] OR "underdeveloped populations"[tw] OR "underdeveloped world"[tw] OR "middle income country"[tw] OR "middle income countries"[tw] OR "middle income nation"[tw] OR "middle income nations"[tw] OR "middle income population"[tw] OR "middle income populations"[tw] OR "low income country"[tw] OR "low income countries"[tw] OR "low income nation"[tw] OR "low income nations"[tw] OR "low income population"[tw] OR "low income populations"[tw] OR "lower income country"[tw] OR "lower income countries"[tw] OR "lower income nation"[tw] OR "lower income nations"[tw] OR "lower income population"[tw] OR "lower income populations"[tw] OR "underserved country"[tw] OR "underserved countries"[tw] OR "underserved nation"[tw] OR "underserved nations"[tw] OR "underserved population"[tw] OR "underserved populations"[tw] OR "underserved world"[tw] OR "underserved country"[tw] OR "underserved countries"[tw] OR "underserved nation"[tw] OR "underserved nations"[tw] OR "underserved population"[tw] OR "underserved populations"[tw]OR "underserved world"[tw] OR "deprived country"[tw] OR "deprived countries"[tw] OR "deprived nation"[tw] OR "deprived nations"[tw] OR "deprived population"[tw] OR "deprived populations"[tw] OR "deprived world"[tw] OR "poor country"[tw] OR "poor countries"[tw]OR "poor nation"[tw] OR "poor nations"[tw] OR "poor population"[tw] OR "poor populations"[tw] OR "poor world"[tw] OR "poorer country"[tw] OR "poorer countries"[tw] OR "poorer nation"[tw] OR "poorer nations"[tw] OR "poorer population"[tw] OR "poorer populations"[tw]OR "poorer world"[tw] OR "developing economy"[tw] OR "developing economies"[tw] OR "less developed economy"[tw] OR "less developed economies"[tw] OR "lesser developed economy"[tw] OR "lesser developed economies"[tw] OR "under developed economy"[tw] OR "underdeveloped economies"[tw] OR "underdeveloped economy"[tw] OR "underdeveloped economies"[tw] OR "middle income economy"[tw] OR "middle income economies"[tw] OR "low income economy"[tw] OR "low income economies"[tw] OR "lower income economy"[tw] OR "lower income economies"[tw] OR "low gdp"[tw] OR "low gnp"[tw] OR "low gross domestic"[tw] OR "low gross national"[tw] OR "lower gdp"[tw] OR "lower gnp"[tw] OR "lower gross domestic"[tw] OR "lower gross national"[tw] OR "lmic"[tw] OR lmics[tw] OR "third world"[tw] OR "lamic country"[tw] OR "lami countries"[tw] OR "transitional country"[tw] OR "transitional countries"[tw] OR Africa[tw] OR Asia[tw] OR Caribbean[tw] OR West Indies[tw] OR South America[tw] OR Latin America[tw] OR Central America[tw] OR Afghanistan[tw] OR Albania[tw]OR Algeria[tw] OR Angola[tw] OR Antigua[tw] OR Barbuda[tw] OR Argentina[tw] OR Armenia[tw] OR Armenian[tw] OR Aruba[tw] OR Azerbaijan[tw] OR Bahrain[tw] OR Bangladesh[tw] OR Barbados[tw] OR Benin[tw] OR Byelarus[tw] OR

For peer review only - http://bmjopen.bmj.com/site/about/guidelines.xhtml

Byelorussian[tw] OR Belarus[tw] OR Belorussian[tw]OR Belorussia[tw] OR Belize[tw] OR Bhutan[tw] OR Bolivia[tw] OR Bosnia[tw] OR Herzegovina[tw] OR Hercegovina[tw] OR Botswana[tw] OR Brasil[tw] OR Brazil[tw] OR Bulgaria[tw] OR Burkina Faso[tw] OR Burkina Fasso[tw] OR Upper Volta[tw] OR Burundi[tw] OR Urundi[tw]OR Cambodia[tw] OR Khmer Republic[tw] OR Kampuchea[tw] OR Cameroon[tw] OR Cameroons[tw] OR Cameron[tw] OR Cape Verde[tw] OR Central African Republic[tw] OR Chad[tw] OR Chile[tw] OR China[tw] OR Colombia[tw] OR Comoros[tw] OR Comoro Islands[tw]OR Comores[tw] OR Mayotte[tw] OR Congo[tw] OR Zaire[tw] OR Costa Rica[tw] OR Cote d'Ivoire[tw] OR Ivory Coast[tw] OR Croatia[tw] OR Cuba[tw] OR Cyprus[tw] OR Czechoslovakia[tw] OR Czech Republic[tw] OR Slovakia[tw] OR Slovak Republic[tw] OR Djibouti[tw] OR French Somaliland[tw] OR Dominica[tw] OR Dominican Republic[tw] OR East Timor[tw] OR East Timur[tw] OR Timor Leste[tw] OR Ecuador[tw] OR Egypt[tw] OR United Arab Republic[tw] OR El Salvador[tw] OR Eritrea[tw] OR Estonia[tw] OR Ethiopia[tw] OR Fiji[tw] OR Gabon[tw]OR Gabonese Republic[tw] OR Gambia[tw] OR Gaza[tw] OR Georgia Republic[tw] OR Georgian Republic[tw] OR Ghana[tw] OR Gold Coast[tw] OR Greece[tw] OR Grenada[tw] OR Guatemala[tw] OR Guinea[tw] OR Guam[tw] OR Guiana[tw] OR Guyana[tw] OR Haiti[tw] OR Honduras[tw]OR Hungary[tw] OR India[tw] OR Maldives[tw] OR Indonesia[tw] OR Iran[tw] OR Iraq[tw] OR Isle of Man[tw] OR Jamaica[tw] OR Jordan[tw] OR Kazakhstan[tw] OR Kazakh[tw] OR Kenya[tw] OR Kiribati[tw] OR Korea[tw] OR Kosovo[tw] OR Kyrgyzstan[tw] OR Kirghizia[tw]OR Kyrgyz Republic[tw] OR Kirghiz[tw] OR Kirgizstan[tw] OR "Lao PDR"[tw] OR Laos[tw] OR Latvia[tw] OR Lebanon[tw] OR Lesotho[tw] OR Basutoland[tw] OR Liberia[tw] OR Libya[tw] OR Lithuania[tw] OR Macedonia[tw] OR Madagascar[tw] OR Malagasy Republic[tw] OR Malaysia[tw]OR Malaya[tw] OR Malay[tw] OR Sabah[tw] OR Sarawak[tw] OR Malawi[tw] OR Nyasaland[tw] OR Mali[tw] OR Malta[tw] OR Marshall Islands[tw] OR Mauritania[tw] OR Mauritius[tw] OR Agalega Islands[tw] OR Mexico[tw] OR Micronesia[tw] OR Middle East[tw] OR Moldova[tw]OR Moldovia[tw] OR Moldovian[tw] OR Mongolia[tw] OR Montenegro[tw] OR Morocco[tw] OR Ifni[tw] OR Mozambique[tw] OR Myanmar[tw] OR Myanma[tw] OR Burma[tw] OR Namibia[tw] OR Nepal[tw] OR Netherlands Antilles[tw] OR New Caledonia[tw] OR Nicaragua[tw] OR Niger[tw]OR Nigeria[tw] OR Northern Mariana Islands[tw] OR Oman[tw] OR Muscat[tw] OR Pakistan[tw] OR Palau[tw] OR Palestine[tw] OR Panama[tw] OR Paraguay[tw] OR Peru[tw] OR Philippines[tw] OR Philipines[tw] OR Phillipines[tw] OR Phillippines[tw] OR Poland[tw] OR Portugal[tw]OR Puerto Rico[tw] OR Romania[tw] OR Rumania[tw] OR Roumania[tw] OR Russia[tw] OR Russian[tw] OR Rwanda[tw] OR Ruanda[tw] OR “Saint Kitts”[tw] OR “St Kitts and Nevis”[tw] OR Saint Lucia[tw] OR St Lucia[tw] OR Saint Vincent[tw] OR St Vincent[tw] OR Grenadines[tw]OR Samoa[tw] OR Samoan Islands[tw] OR Navigator Island[tw] OR Navigator Islands[tw] OR Sao Tome[tw] OR Saudi Arabia[tw] OR Senegal[tw] OR Serbia[tw] OR Montenegro[tw] OR Seychelles[tw] OR Sierra Leone[tw] OR Slovenia[tw] OR Sri Lanka[tw] OR Ceylon[tw] OR Solomon Islands[tw] OR Somalia[tw] OR Sudan[tw] OR Suriname[tw] OR Surinam[tw] OR Swaziland[tw] OR Syria[tw] OR Tajikistan[tw] OR Tadzhikistan[tw] OR Tadjikistan[tw] OR Tadzhik[tw] OR Tanzania[tw] OR Thailand[tw] OR Togo[tw] OR Togolese Republic[tw] OR Tonga[tw] OR Trinidad[tw] OR Tobago[tw] OR Tunisia[tw] OR Turkey[tw] OR Turkmenistan[tw] OR Turkmen[tw] OR Uganda[tw] OR Ukraine[tw] OR Uruguay[tw] OR USSR[tw] OR Soviet Union[tw] OR Union of Soviet Socialist Republics[tw] OR Uzbekistan[tw] OR Uzbek OR Vanuatu[tw] OR New Hebrides[tw] OR Venezuela[tw] OR Vietnam[tw] OR Viet Nam[tw] OR West Bank[tw] OR Yemen[tw] OR Yugoslavia[tw] OR Zambia[tw] OR Zimbabwe[tw] OR Rhodesia[tw] OR Developing Countries[Mesh:noexp] OR Africa[Mesh:noexp] OR Africa, Northern[Mesh:noexp] OR Africa South of the Sahara[Mesh:noexp] OR Africa, Central[Mesh:noexp] OR Africa, Eastern[Mesh:noexp] OR Africa, Southern[Mesh:noexp] OR Africa, Western[Mesh:noexp] OR Asia[Mesh:noexp] OR Asia, Central[Mesh:noexp] OR Asia, Southeastern[Mesh:noexp] OR Asia, Western[Mesh:noexp]OR Caribbean Region[Mesh:noexp] OR West Indies[Mesh:noexp] OR South America[Mesh:noexp] OR Latin America[Mesh:noexp] OR Central America[Mesh:noexp] OR Afghanistan[Mesh:noexp] OR Albania[Mesh:noexp] OR Algeria[Mesh:noexp] OR American Samoa[Mesh:noexp] OR Angola[Mesh:noexp]OR "Antigua and Barbuda"[Mesh:noexp] OR Argentina[Mesh:noexp] OR Armenia[Mesh:noexp] OR Azerbaijan[Mesh:noexp] OR Bahrain[Mesh:noexp] OR Bangladesh[Mesh:noexp] OR Barbados[Mesh:noexp] OR Benin[Mesh:noexp] OR Byelarus[Mesh:noexp] OR Belize[Mesh:noexp] OR Bhutan[Mesh:noexp]OR Bolivia[Mesh:noexp] OR Bosnia-Herzegovina[Mesh:noexp] OR Botswana[Mesh:noexp] OR Brazil[Mesh:noexp] OR Bulgaria[Mesh:noexp] OR Burkina Faso[Mesh:noexp] OR Burundi[Mesh:noexp] OR Cambodia[Mesh:noexp] OR Cameroon[Mesh:noexp] OR Cape Verde[Mesh:noexp] OR Central African Republic[Mesh:noexp] OR Chad[Mesh:noexp] OR Chile[Mesh:noexp] OR China[Mesh:noexp] OR Colombia[Mesh:noexp] OR Comoros[Mesh:noexp] OR Congo[Mesh:noexp] OR Costa Rica[Mesh:noexp] OR Cote d'Ivoire[Mesh:noexp] OR Croatia[Mesh:noexp] OR Cuba[Mesh:noexp]OR Cyprus[Mesh:noexp] OR Czechoslovakia[Mesh:noexp] OR Czech Republic[Mesh:noexp] OR Slovakia[Mesh:noexp] OR Djibouti[Mesh:noexp] OR "Democratic Republic of the Congo"[Mesh:noexp] OR Dominica[Mesh:noexp] OR Dominican Republic[Mesh:noexp] OR East Timor[Mesh:noexp]OR Ecuador[Mesh:noexp] OR Egypt[Mesh:noexp] OR El Salvador[Mesh:noexp] OR Eritrea[Mesh:noexp] OR Estonia[Mesh:noexp] OR Ethiopia[Mesh:noexp] OR Fiji[Mesh:noexp] OR Gabon[Mesh:noexp] OR Gambia[Mesh:noexp] OR "Georgia (Republic)"[Mesh:noexp] OR Ghana[Mesh:noexp]OR Greece[Mesh:noexp] OR Grenada[Mesh:noexp] OR Guatemala[Mesh:noexp] OR Guinea[Mesh:noexp] OR Guinea-Bissau[Mesh:noexp] OR Guam[Mesh:noexp] OR Guyana[Mesh:noexp] OR Haiti[Mesh:noexp] OR Honduras[Mesh:noexp] OR Hungary[Mesh:noexp] OR India[Mesh:noexp] OR Indonesia[Mesh:noexp]OR Iran[Mesh:noexp] OR Iraq[Mesh:noexp] OR Jamaica[Mesh:noexp] OR Jordan[Mesh:noexp] OR Kazakhstan[Mesh:noexp] OR Kenya[Mesh:noexp] OR Korea[Mesh:noexp] OR Kosovo[Mesh:noexp] OR Kyrgyzstan[Mesh:noexp] OR Laos[Mesh:noexp] OR Latvia[Mesh:noexp] OR Lebanon[Mesh:noexp]OR Lesotho[Mesh:noexp] OR Liberia[Mesh:noexp] OR Libya[Mesh:noexp] OR Lithuania[Mesh:noexp] OR Macedonia[Mesh:noexp] OR Madagascar[Mesh:noexp] OR Malaysia[Mesh:noexp] OR Malawi[Mesh:noexp] OR Mali[Mesh:noexp] OR Malta[Mesh:noexp] OR Mauritania[Mesh:noexp]OR Mauritius[Mesh:noexp] OR Mexico[Mesh:noexp] OR Micronesia[Mesh:noexp] OR Middle East[Mesh:noexp] OR Moldova[Mesh:noexp] OR Mongolia[Mesh:noexp] OR Montenegro[Mesh:noexp] OR Morocco[Mesh:noexp] OR Mozambique[Mesh:noexp] OR Myanmar[Mesh:noexp] OR Namibia[Mesh:noexp]OR Nepal[Mesh:noexp] OR Netherlands Antilles[Mesh:noexp] OR New Caledonia[Mesh:noexp] OR Nicaragua[Mesh:noexp] OR Niger[Mesh:noexp] OR Nigeria[Mesh:noexp] OR Oman[Mesh:noexp] OR Pakistan[Mesh:noexp] OR Palau[Mesh:noexp] OR Panama[Mesh:noexp] OR Papua New Guinea[Mesh:noexp]OR Paraguay[Mesh:noexp] OR Peru[Mesh:noexp] OR Philippines[Mesh:noexp] OR Poland[Mesh:noexp] OR Portugal[Mesh:noexp] OR Puerto Rico[Mesh:noexp] OR Romania[Mesh:noexp] OR Russia[Mesh:noexp] OR "Russia (Pre-1917)"[Mesh:noexp] OR Rwanda[Mesh:noexp] OR "Saint Kitts and Nevis"[Mesh:noexp] OR Saint Lucia[Mesh:noexp] OR "Saint Vincent and the Grenadines"[Mesh:noexp] OR Samoa[Mesh:noexp] OR Saudi Arabia[Mesh:noexp] OR Senegal[Mesh:noexp] OR Serbia[Mesh:noexp] OR Montenegro[Mesh:noexp] OR Seychelles[Mesh:noexp] OR Sierra Leone[Mesh:noexp] OR Slovenia[Mesh:noexp] OR Sri Lanka[Mesh:noexp] OR Somalia[Mesh:noexp] OR South Africa[Mesh:noexp] OR Sudan[Mesh:noexp] OR Suriname[Mesh:noexp] OR Swaziland[Mesh:noexp] OR Syria[Mesh:noexp] OR Tajikistan[Mesh:noexp] OR Tanzania[Mesh:noexp]OR

For peer review only - http://bmjopen.bmj.com/site/about/guidelines.xhtml

Thailand[Mesh:noexp] OR Togo[Mesh:noexp] OR Tonga[Mesh:noexp] OR "Trinidad and Tobago"[Mesh:noexp] OR Tunisia[Mesh:noexp] OR Turkey[Mesh:noexp] OR Turkmenistan[Mesh:noexp] OR Uganda[Mesh:noexp] OR Ukraine[Mesh:noexp] OR Uruguay[Mesh:noexp] OR USSR[Mesh:noexp]OR Uzbekistan[Mesh:noexp] OR Vanuatu[Mesh:noexp] OR Venezuela[Mesh:noexp] OR Vietnam[Mesh:noexp] OR Yemen[Mesh:noexp] OR Yugoslavia[Mesh:noexp] OR Zambia[Mesh:noexp] OR Zimbabwe[Mesh:noexp])

For peer review only

Appendix 2. Extraction sheet

Appendix 2, Extraction terms	
**Background details**	First author surname<br>Year<br>Title<br>Country<br>Region<br>Intervention- Funding source<br>Evaluation- Funding source<br>Author email address
**Study Context**	Study location:<br>rural=1;<br>urban=2;<br>peri-urban=3;<br>mixed (urban/rural/peri-urban)=4<br>Background and history of groups<br>(who formed, trained and maintained the groups in the study areas)<br>Health system strength/indicators<br>(in author's words- the health systems setting, the important health indicators talked about in the study area)<br>Specific policy environment towards groups (NRLM, linkages with other devlopment programmes)<br>Others
**Hypothesis tested |&| Theory of Change**	What is the underlying hypothesis being tested in the paper?<br>What is the implicit or explicit theory of change?<br>Were any statistical analyses done to test the TOC?<br>Yes/ No- 1/0<br>What are the effect modifiers/ anticipated effect modifiers, if any<br>What SBC theory is used implicitly or explicitly?
**Intervention**	Describe the intervention and its components in the authors' own words<br>Intervention description- On field implementation details (Text from paper/ rewrite in brief)<br>What elements of the intervention were participatory (in authors' own words)<br>What is the average group size?<br>Who was the facilitator?<br>(1- Internal member, trained from within the group; 2- External facilitator, from NGO or Govt.)<br>Facilitator characteristics (education, residence, social group, sex, etc).<br>What training was given to the facilitator in authors' own words?<br>Were the facilitators given any monetary incentive? Y=1, N=0<br>Number of groups per facilitator (con)

	Number of groups per facilitator (exp)<br>intervention period?<br>Number of sessions<br>Is this a mixed method evaluation? Yes=1, No=2<br>Level of participation of members in intervention design and implementation (Arnstein): Informed/Consulted/Partners<br>Level of capability strengthening for health (Labonte and Laverack): Individual/Group/community/other<br>Intervention communication approach: Didactic/Interactive/Participatory<br>Category of group intervention (Gram et al): Class/Club/Collective
**Group details**	How often did groups meet?<br>Underlying group type:-<br>1= Microfinance groups<br>2= Open groups<br>3= Community based women only groups<br>4= Special population groups<br>Average age (and range) of members in groups<br>What is the duration of each meeting?<br>Population coverage of groups? (% coverage)<br>Group dissolution during the intervention period<br>What are the service linkages (health specific), if any?
**Intensity/ Coverage of Intervention**	Details on functioning of Groups in Control arm<br>Details on functioning of Groups in Intervention arm<br>Topics discussed in a regular (control) group meeting + length of discussion<br>Additional topics discussed in Intervention arm group meetings (Due to layering) + length of discussion<br>Frequency of add-on intervention<br>% members who attended the add-on intervention sessions<br>Exposure to the intervention (text from paper, if any)<br>Target group of intervention - does this align with group membership (age, demographic characteristics). In authors words if available
**Participant Inclusion Criterion**	Evaluation-<br>Participant eligibility, inclusion criteria [text]<br>Evaluation-<br>Participant eligibility, Exclusion criteria [text]<br>Intervention-<br>Participant eligibility, inclusion criteria [text]<br>Intervention-<br>Participant eligibility, Exclusion criteria [text]
**Evaluation details**	How would you characterise the evaluation on the efficacy/effectiveness spectrum?

	1 = towards efficacy (implementation on small/medium scale by non-gov partners);<br>2 = implementation on small/medium scale by gov partners in conditions close to routine;<br>3 = implementation on large scale, through routine training systems by gov partners<br>Evaluation design (1= RCT,<br>2=Longitudinal Cohort,<br>3= Repeated Cross-sectional survey<br>4= Pre/ post evaluation design<br>5=Other (specify, eg. Type of Quasi experimental)<br>Nature of comparison (1=exp vs con; 2=before vs after intervention)<br>Is there a control group? Yes=1, No=2<br>Type of control (group with no intervention OR no group, with or without matching)<br>Is this a cross-sectional baseline/endline survey design? Yes=1, No=2<br>Please describe survey design [text]
**Cost effectiveness component**	Does the study include a cost effectiveness component?<br>Yes--1<br>No--2<br>Is the cost given a total cost figure, or separate cost given for each descriptive component of the intervention?<br>Total cost--1<br>Individual cost components--2<br>Cost effectiveness method used (text from paper)<br>Cost value (figure given in the paper with unit)
**Outcome areas**	MNCH<br>SRH and HIV<br>Family planning<br>General health/morbidity<br>Violence<br>Mental health<br>Nutrition<br>(Includes Breastfeeding)<br>Non-communicable diseases<br>Communicable diseases<br>Expenditure<br>Health insurance<br>Sanitation<br>Outcomes at group level or population level?<br>1- Group level<br>2- Population/ Community level<br>3. Sample members and non-members (from the community)<br>Non health outcomes, if any?<br>What is the primary outcome?<br>What are the secondary outcomes?<br>Does study differentiate between primary and secondary outcomes? Yes=1/no=0

**Timing of data collection**	baseline<br>endline<br>Time between baseline and endline<br>other information (if any, write it in text)
**Data collection and statistical methods**	Sampling methods in the authors' own words<br>Is the analysis by Intention-to-treat> (yes=1, no=0)<br>Confounders assessed/adjusted for?<br>Unit of clustering<br><br>Clustering at --<br>1: geographical level, 2: clusters formed using specific characteristics (other than geography)<br>Number and size of clusters<br>Adjusted for clustering in analysis? Yes=1/no=0<br>Does the study have a sample size calculation? Yes=1, No=0<br>Does the study have a published protocol or registration with details of outcomes and analysis methods? Yes=1, No=0<br>Randomisation method, if any<br>Blinding process, in authors' own words (i.e. were any or all of persons who did random allocation, data collectors, and analysts blind to exposure/cont rolallocation?).<br>N eligible participants (exp vs control or before/after)<br>Loss to follow-up/Attrition (individual)<br>Method of assessment (write name of questionnaire/describe method)<br>e.g specific scales used like PHQ, CESD etc or anthropometric or biometric measurements taken<br>Statistical method (the name of analysis)
**Results**	mean age- separate baseline and endline if appropriate<br>Literacy - separate baseline and endline if appropriate<br>% of participants belonging to marginalised groups (con) - separate baseline and endline if appropriate
	N eligible participants (exp vs control or before/after)
**Results: Primary Outcomes**	Results - Mean (SD) in exp/con or before/after participants<br>Results - Rate in exp/con or before/after participants<br>Results - Prevalence in exp/con or before/after participants<br>Results - incidence in exp/con or before/after participants<br>Results -Odds Ratio (95% CI) for exp/con or before/after participants specify adjusted and unadjusted<br>Results - Rate ratio (95% CI) for exp/con or before/after participants specify adjusted and unadjusted<br>Results; Beta coefficient (95% CI) for exp/con or before/after participants specify adjusted and unadjusted<br>Results: difference-in-difference (DID) (difference in % points)- control v/s intervention before vs after
	Results; P-value for difference between groups
**Results: Secondary Outcomes**	Results: report all results on secondary outcomes with effect sizes (95% CI) and p-value - use ITT results, not sub-group analyses -

For peer review only - http://bmjopen.bmj.com/site/about/guidelines.xhtml

	Highlight significant findings (CI does not include 1) in red or with asterisk<br>Notes on secondary outcomes
**Qualitative Results and Appraisal**	Qualitative sample<br>Qualitative data collection methods<br>Qualitative results<br>Clarity of the research question<br>Explanation of the context<br>Sampling<br>Reflexivity of the account<br>Clear account of the methods used for data collection and analysis<br>Amount of data pertinent to the review question
**Authors' Own Account Of Study Limitations**	Notes on bias
**Risk of Bias assessment**	ROB-2 and ROBINS
**Barriers And Enablers To Intervention Achieving Impact**	Enablers for the intervention in the authors words<br>Barriers for the intervention in the authors words<br>Reviewers interpretation of barriers and enablers.
**Layering specific Barriers and Enablers**	Barriers to layering the intervention<br>Enablers to layering the intervention
**Process Evaluation**	Does this study include a process evaluation<br>Is the process evaluation quantitative, qualitative or mixed methods (give citation here)
**Additional information, if any**	Notes/ Remarks

For peer review only - http://bmjopen.bmj.com/site/about/guidelines.xhtml

For peer review only

For peer review only - http://bmjopen.bmj.com/site/about/guidelines.xhtml

PRISMA-P (Preferred Reporting Items for Systematic review and Meta-Analysis Protocols) 2015 checklist: recommended items to address in a systematic review protocol*

Section and topic	Item No	Checklist item	Page number
**ADMINISTRATIVE INFORMATION**			
Identification	1a	Identify the report as a protocol of a systematic review	Title, p. 1; abstract, p. 2; Introduction, p. 4
Update	1b	If the protocol is for an update of a previous systematic review, identify as such	Not an update
Registration	2	If registered, provide the name of the registry (such as PROSPERO) and registration number	Abstract, p. 3, PROSPERO registration number provided
Authors:			
Contact	3a	Provide name, institutional affiliation, e-mail address of all protocol authors; provide physical mailing address of corresponding author	Title page, p. 1
Contributions	3b	Describe contributions of protocol authors and identify the guarantor of the review	Author contributions, p. 13
Amendments	4	If the protocol represents an amendment of a previously completed or published protocol, identify as such and list changes; otherwise, state plan for documenting important protocol amendments	Plans to report protocol amendments noted in Ethics and Dissemination, p. 11
Support:			
Sources	5a	Indicate sources of financial or other support for the review	Funding statement, p. 13
Sponsor	5b	Provide name for the review funder and/or sponsor	
Role of sponsor or funder	5c	Describe roles of funder(s), sponsor(s), and/or institution(s), if any, in developing the protocol	Funding statement, p. 13
INTRODUCTION			
Describe the rationale for the review in the context of what is already known			Introduction, pp. 4-5
Objectives	7	Provide an explicit statement of the question(s) the review will address with reference to participants, interventions, comparators, and outcomes (PICO)	Introduction, p. 5
METHODS			
Eligibility criteria: Specify the study characteristics (such as PICO, study design, setting, time frame) and report characteristics (such as years considered, language, publication status) to be used as criteria for eligibility for the review			Study design, Participants, Types of Interventions, pp. 5-7

For peer review only

Information sources	9	Describe all intended information sources (such as electronic databases, contact with study authors, trial registers or other grey literature sources) with planned dates of coverage	Information sources, p. 8
Search strategy	10	Present draft of search strategy to be used for at least one electronic database, including planned limits, such that it could be repeated	Search strategy, p. 8; Appendix 1
Study records:			
Data management	11a	Describe the mechanism(s) that will be used to manage records and data throughout the review	Data management, pp. 8-9
Selection process	11b	State the process that will be used for selecting studies (such as two independent reviewers) through each phase of the review (that is, screening, eligibility and inclusion in meta-analysis)	Selection strategy, p. 9
Data collection process	11c	Describe planned method of extracting data from reports (such as piloting forms, done independently, in duplicate), any processes for obtaining and confirming data from investigators	Data collection process, p. 9
Data items	12	List and define all variables for which data will be sought (such as PICO items, funding sources), any pre-planned data assumptions and simplifications	Data items, pp. 9-10; Appendix 2
Outcomes and prioritization	13	List and define all outcomes for which data will be sought, including prioritization of main and additional outcomes, with rationale	Outcomes, pp. 7-8; Outcomes and prioritisation, p. 10
Risk of bias in individual studies	14	Describe anticipated methods for assessing risk of bias of individual studies, including whether this will be done at the outcome or study level, or both; state how this information will be used in data synthesis	Risk of bias in individual studies, p. 10
Data synthesis	15a	Describe criteria under which study data will be quantitatively synthesised	Data synthesis, pp. 10-11
Data synthesis Meta-bias(es)	15b	If data are appropriate for quantitative synthesis, describe planned summary measures, methods of handling data and methods of combining data from studies, including any planned exploration of consistency (such as I^2, Kendall's τ)	Data synthesis, pp. 10-11
	15c	Describe any proposed additional analyses (such as sensitivity or subgroup analyses, meta-regression)	N/A
	15d	If quantitative synthesis is not appropriate, describe the type of summary planned	Data synthesis, pp. 10-11
	16	Specify any planned assessment of meta-bias(es) (such as publication bias across studies, selective reporting within studies)	Meta-biases, p. 11
Confidence in cumulative evidence	17	Describe how the strength of the body of evidence will be assessed (such as GRADE)	Confidence in cumulative evidence, p. 11

For peer review only

*** It is strongly recommended that this checklist be read in conjunction with the PRISMA-P Explanation and Elaboration (cite when available) for important clarification on the items. Amendments to a review protocol should be tracked and dated. The copyright for PRISMA-P (including checklist) is held by the PRISMA-P Group and is distributed under a Creative Commons Attribution Licence 4.0.**

From: Shamseer L, Moher D, Clarke M, Ghersi D, Liberati A, Petticrew M, Shekelle P, Stewart L, PRISMA-P Group. Preferred reporting items for systematic review and meta-analysis protocols (PRISMA-P) 2015: elaboration and explanation. BMJ. 2015 Jan 2;349(jan02 1):g7647.

For peer review only
